# Evolutionary Analysis of MBW Function by Phenotypic Rescue in *Arabidopsis thaliana*

**DOI:** 10.3389/fpls.2019.00375

**Published:** 2019-03-29

**Authors:** Bipei Zhang, Martin Hülskamp

**Affiliations:** Botanical Institute, Biocenter, University of Cologne, Cologne, Germany

**Keywords:** MBW complex, protein function, *Arabidopsis thaliana*, *Arabis alpina*, *Gossypium hirsutum*, *Petunia hybrida*, *Zea mays*

## Abstract

The MBW complex consisting of the three proteins R2R3MYB, bHLH and WDR regulates five traits in *Arabidopsis thaliana* including trichome and root hair patterning, seed coat color, anthocyanidin production and seed coat mucilage release. The WDR gene *TTG1* regulates each trait in specific combinations with different bHLH and R2R3MYB proteins. In this study we analyze to what extent the biochemical properties of the MBW proteins contribute to trait specificity by expressing them in appropriate *A. thaliana* mutants. We show that the rescue behavior of *A. thaliana* bHLH and R2R3MYB protein is sufficient to explain the function as derived previously from mutant analysis. When extending this rescue approach using MBW proteins from other species we find that proteins involved in anthocyanidin regulation typically show a rescue of the anthocyanidin phenotype but not of the other traits. Finally, we correlate the rescue abilities of MBW protein from different species with the *A. thaliana* proteins.

## Introduction

In the model plant *Arabidopsis thaliana* five traits including trichome and root hair patterning, seed coat color, anthocyanidin production and seed coat mucilage release are genetically linked through the action of the *TRANSPARENT TESTA GLABRA1* (*TTG1*) gene (Koornneef, 1981). Mutations in the *TTG1* gene strongly affect all five traits. *TTG1* encodes a WDR protein (Walker et al., 1999) that forms protein complexes with R2R3MYB and basic Helix-Loop-Helix factors (bHLH) that transcriptionally regulate downstream target genes (for review see Lloyd et al., 2017). This complex is called the MBW complex (Ramsay and Glover, 2005; Xu et al., 2015; Zhang and Schrader, 2017) and it forms through the binding of TTG1 and the MYB protein to the bHLH protein (Payne et al., 2000; Zhang et al., 2003; Zimmermann et al., 2004; Feller et al., 2006). In addition, most bHLH proteins dimerise suggesting that higher ordered protein complexes are possible (Payne et al., 2000; Bernhardt et al., 2003; Zhang et al., 2003). While *TTG1* is a single copy gene, there are four bHLH genes including *GL3* (Hülskamp et al., 1994; Payne et al., 2000; Bernhardt et al., 2003; Zhang et al., 2003; Gonzalez et al., 2008; Feyissa et al., 2009), *EGL3* (Bernhardt et al., 2003; Zhang et al., 2003; Gonzalez et al., 2008), *MYC1* (Symonds et al., 2011; Bruex et al., 2012) and *TT8* (Nesi et al., 2000; Zhang et al., 2003; Baudry et al., 2006) involved. The genetic analysis revealed some trait specificity such that each bHLH is genetically important for two or more of the five traits. The R2R3MYB genes involved in the regulation of the five *TTG1*-dependent traits are represented by several genes. These display high trait specificity. Trichome patterning is regulated by *GL1* and *MYB23* (Herman and Marks, 1989; Marks and Feldmann, 1989; Oppenheimer et al., 1991; Kirik et al., 2005), root hair patterning by *WER* (Lee and Schiefelbein, 1999), seed coat mucilage by *MYB61* (Penfield et al., 2001; Zhang et al., 2003) and MYB5 (Gonzalez et al., 2009), seed color (proanthocyanidin production) by *TT2* (Baudry et al., 2004; Gonzalez et al., 2009) and anthocyanidin by *PAP1* and *PAP2* (Borevitz et al., 2000; Teng et al., 2005; Gonzalez et al., 2008). Taken together these findings led to the concept that different MBW complexes discriminate between different traits. This raises the question, whether this specificity is due to different protein functions or different transcriptional regulation. On the one hand, target gene specificity has been mapped to a few relevant amino acids in the MYB proteins TT2 and PAP4 highlighting that specificity is mediated at the level of protein functions (Heppel et al., 2013; Lai et al., 2013). On the other hand, there is some evidence available for R2R3MYB proteins suggesting that the proteins have equivalent functions and that differences in the transcriptional regulation lead to the observed genetic specificity. *GL1* and *WER* are excellent examples as GL1 expressed under the *WER* promoter can rescue the *wer* root hair phenotype and *vice versa* (Lee and Schiefelbein, 2001). The bHLH proteins may have properties that are relevant for the specific traits. This is exemplified by the ability of GL3 and EGL3 to rescue *myc1* mutants under the MYC1 promoter whereas MYC1 cannot rescue *gl3 egl3* mutants under the *GL3* or the *EGL3* promoters (Zhao et al., 2012). Thus, there is evidence available for both possibilities, specificity by transcriptional regulation and protein specificity (Lai et al., 2013; Lloyd et al., 2017).

The MBW network is evolutionary conserved in plants and was reported in monocots, dicots, angiosperms and gymnosperms (Ramsay and Glover, 2005; Quattrocchio et al., 2006a; Serna and Martin, 2006; Xu et al., 2015; Nemesio-Gorriz et al., 2017; Zhang and Schrader, 2017). The regulation of the flavonoid pathway is considered to be the most ancient role of this complex. It is postulated that additional functions have evolved by gene duplications followed by functional diversification after rosid-asterid split (Serna and Martin, 2006). Consistent with this, mutations in the WDR gene *TTG1* in *Arabis alpina* and *Matthiola incana* lead to phenotypes in all five traits affected in *A. thaliana* (Koornneef, 1981; Dressel and Hemleben, 2009; Chopra et al., 2014). Functional conservation between Arabidopsis and more distantly related species were frequently assessed by rescuing the respective Arabidopsis mutants by overexpressing genes under ubiquitous promoters or gene-specific promoters (see references in Supplementary Table S1 for details).

In this study, we aimed to understand to what extent MBW protein function is relevant for rescuing each of the five *TTG1*-dependent traits in Arabidopsis mutants. Toward this end, we expressed the MBW genes under the 35S promoter in the respective mutants and compare our results to previously published results. In a first step, we studied Arabidopsis genes. For bHLH genes we show that their functional specialization can be explained by specialized protein function. In a second step, we analyzed MBW proteins from other species including *Arabis alpina*, cotton (*Gossypium hirsutum*), petunia (*Petunia x hybrida*) and maize (*Zea mays*). Finally, we assessed the ability of MBW proteins from other species to interact with Arabidopsis MBW proteins to judge the relevance of changes in the protein–protein interactions for rescue ability.

## Materials and Methods

### Arabidopsis Strains and Plant Growth

The mutant alleles used in this study: *ttg1-1* (*Ler*) (Koornneef, 1981; Walker et al., 1999). *gl3-3* (*Col*) background (Jakoby et al., 2008); *egl3-77439* (*Col*) background (TAIR accession 1008704039); *tt8-048673* (*Col*) background (TAIR accession 1005848854); *gl3 egl3* (*Col*) (Friede et al., 2017); *gl3/egl3/tt8* (homozygous progeny by crossing *gl3/egl3* with *tt8-048673*); *gl1* (*Col*) (Oppenheimer et al., 1991); *pap1* (pst16228) (No-0; CS28564) (Kuromori et al., 2004); *pap2* (*Col*) (salk_093731, TAIR accession 1005457343). Plants were grown on soil at 24°C with 16 h of light per day. All transgenic plant lines used and created in this work are listed in Supplementary Table S2. Transgenic lines were generated using the floral dip method (Clough and Bent, 1998). Transgenic T1 plants were selected on ½ MS agar plates containing 10 mg/L BASTA [glufosinate ammonium, Sigma-Aldrich (Munic, Steinheim)].

### Constructs

All CDS entry clones were generated by amplifying the CDSs from start to stop codon from *A. thaliana* (*At*), *Arabis alpina* (*Aa*), *G. hirsutum* (*Gh*), *Petunia hybrid* (*Ph*), and *Zea mays* (*Zm*) followed by BP recombination in pDONR201/207.

### LUMIER Vectors

Three different destination vectors were used for subsequent LR reactions. pcDNA3-Rluc-GW and pTREX-dest30 (Invitrogen) for the N-terminal fusion of *Renilla reniformis* and *Staphylococcus aureus* protein, respectively, were described before (Pesch et al., 2015).

Genes were N-terminal fused to the *S. aureus* protein A sequence in pTREX-dest30-ntProtA by LR reaction. For the negative control, the vector pTREX-dest30-ntProtA was recombined with pENTR1A-w/occdB.

The full-length Renilla luciferase sequence was N-terminal fused to the coding sequences in pcDNA3-Rluc-GW. Also pENTR1A-w/o-ccdB was recombined to this vector as a negative control.

### Plant Vectors

All CDS of MBW homologs were cloned into Donor vectors by BP reactions (Invitrogen). Then recombination of the corresponding entry clones with the 35S promoter containing vector pAMPAT-35S-GW [GenBank accession no. AY436765 (Pesch et al., 2015)].

### Phenotypic Analysis

The presence of trichomes was scored on leaves 1–6 on 2-week-old seedlings. For the analysis of root hairs H-files were identified microscopically by their position over cortical cell boundaries in roots of 7-day-old 1/2MS plate-grown T2 progeny seedlings The number root hairs in the flanking N-files was roughly determined in 1-week-old basta-resistant T2 seedlings under a Leica stereomicroscope (MZ FLIII).

Anthocyanidin was analyzed in T2 seedlings grown on 1/2MS germination medium containing 3% sucrose, and analyzed using a Leica stereomicroscope (MZ FLIII) with the Multi-Focus and Montage option of the Leica Application Suite V3 (Leica Microsystems, Wetzlar, Germany). Seed coat color was observed in T1 progeny seeds and pictures were taken with a Canon EOS 5D Mark (Canon, Krefeld, Germany). For the analysis of seed mucilage T2 seeds were imbibed in 0.2% w/v aqueous ruthenium red (Sigma) solution with 0.5% agar and monitored after 3–4 h by light microscopy and pictures taken using the DISKUS software (Carl H. Hilgers Technisches Büro, Germany) (Modified from Penfield et al., 2001).

### LUMIER (LUminescence-Based Mammalian IntERactome)

For LUMIER assays, each protein was transiently expressed in HEK293TM cells (BioCat/SBI: LV900A-1) as hybrid proteins either with the *S. aureus* protein A (ProtA) or with the *R. reniformis* luciferase (Rluc) fused to their amino N termini. The LUMIER assay is based on the technique established for high throughput screens (Blasche and Koegl, 2013) and was optimized for the proteins used in this study (Pesch et al., 2013; Pesch et al., 2015). Constructs (1.5 μg plasmid DNA) were single-transfected into 1 × 10^6^ HEK293TM cells in 6-well plates using 10 μl Lipofectamine 2000 (Thermo). After 48 h, the medium was removed and cells were washed with PBS. The cells were collected by centrifugation (600 g, 15 min) and lysed on ice in 50 μl ice-cold lysis buffer [20 mM Tris pH 7.5, 250 mM NaCl, 1.1% Triton X-100, 10 mM EDTA, 10 mM DTT, Protease Inhibitor Cocktail (Roche, 1836170) and benzonase (Novagen, 70746; 0.0125 units per μl final concentration)] for 1 h. Subsequently, lysates were centrifuged at 15,000 rpm (21,380 g) for 15 min at 4°C, and the supernatant was mixed with 10 μl PBS-prewashed sheep anti-rabbit IgG-coated magnetic beads (Invitrogen, Dynabeads M280; 2 mg/ml final concentration) and incubated for 1 h on ice. The beads were collected using a magnetic holder (Neodym-Magnets, 1.3 Teslar) and luminescence measured in a microtiter plate reader (BMG Fluostar Optima). The pulldown was also performed with untransfected cells and with cells solely expressing Luciferase-protein to exclude any non-specific signal from the cell lysate and non-specific binding of Luciferase-protein to the beads, respectively.

The percentage of Rluc on the beads compared with the lysate was calculated by dividing the Rluc activity on the beads by the Rluc activity in the same amount of lysate used in the pull-down assay (Input). Pulldown efficiency was calculated by: [Rluc pulldown/Input] × 100.

### Phylogenetic Tree

Unrooted Maximum Likelihood Phylogenetic Trees for WDR, bHLH, and R2R3MYB was done with MEGA6 (Tamura et al., 2013) using entire amino acid sequences based on the JTT matrix-based model (Jones et al., 1992). In addition to the proteins used in this study we added proteins from other species also discussed to play a role in anthocyanidin regulation (Zhang and Schrader, 2017).

## Results

In order to study the functional conservation of MBW proteins we performed rescue experiments in suitable *A. thaliana* mutants under the 35S promoter. We used the same strong promoter for all experiments to facilitate a direct comparison of the protein properties in all experiments. Although this strategy has the disadvantage that our results may differ to published experiments using gene-specific promoters (Supplementary Table S1) we consider this to be a reasonable procedure to compare the protein properties. For completeness and for comparison in our assay system we also included genes for which this type of rescue data were already published.

**Figure 1:**
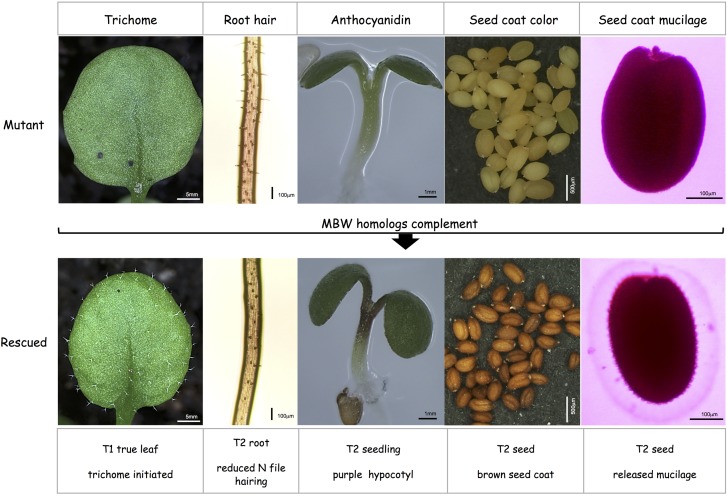
Rescue strategy for the five TTG1-dependent traits in *Arabidopsis* mutants. **(Top row)** *ttg1* mutant or *gl3 egl3 tt8* triple mutant phenotypes display glabrous rosette leaves, extra root hairs in N-files, no anthocyanidin production under stress conditions in the hypocotyl, yellow seeds and no seed coat mucilage release. **(Bottom row)** rescue by the expression of MBW homologs: recovery of trichomes, suppression of extra root hairs in N-files, purple hypocotyls under stress conditions, brown seeds and the release of seed coat mucilage (light pink Ruthenium Red labeled region around the seed).

Rescue of all five traits were analyzed qualitatively in the T1 generation (Figure 1) to judge the rescue efficiency at different chromosomal insertions. Trichomes were scored on rosette leaves of 3-week-old seedlings. The pro-anthocyanidin and seed coat mucilage phenotype were assayed in T2 seeds because both are maternal traits. Also anthocyanidin production and root hairs were studied in the T2 generation for technical reasons. However, rescue was still compared at the level of the T1 generation as represented by analyzed T2 plants. We studied anthocyanidin production in the hypocotyl on 4 days old seedlings grown on MS with 3% sugar by visual inspection (Teng et al., 2005). Seed coat mucilage was analyzed by staining the mucilage with Ruthenium Red (Penfield et al., 2001). Pro-anthocyanidin production was inspected by seed color and root hairs by assessing trichomes in N-file files of 1-week-old basta-resistant T2 seedlings.

For our functional comparison we initially tested the Arabidopsis MBW proteins for their ability to rescue the different traits. Then we selected MBW proteins from other species including *A. alpina*, cotton (*Gossypium hirsutum*), petunia (*Petunia x hybrida*) and maize (*Z. mays*). Species and genes were chosen based on already published information suggesting that the MBW genes are relevant in the regulation of the traits considered here. The phylogenetic relationship of the proteins is shown in Supplementary Figure S1.

### *Rescue* of the Arabidopsis *ttg1* Mutant by AtTTG1 Homologs

In order to analyze whether the protein function of homologous *TTG1* genes in other species is conserved we performed rescue experiments in *A. thaliana ttg1-1* mutants (Koornneef, 1981; Walker et al., 1999). Toward this end we selected *TTG1* homologs from several species including *A. thaliana* (*AtTTG1*), *A. alpina* (*AaTTG1*), *G. hirsutum* (*GhTTG1*, *GhTTG2*, *GhTTG3*, *GhTTG4*), *P. hybrida* (*PhAN11*) and *Z. mays* (*ZmPAC1*, *ZmMP1*). *A. thaliana TTG1* served as a control for the rescue efficiency.

*Arabidopsis thaliana* and *A. alpina* TTG1 showed efficient rescue of all traits in most T1 lines (Table 1). The cotton GhTTG1 and GhTTG3 proteins also rescued all traits in more than 50% of the T1 lines [see also (Humphries et al., 2005)]. GhTTG2 and GhTTG4 did not show rescue which is consistent with the previous result that transient expression cannot rescue the anthocyanidin phenotype of the *ttg1* mutation in *M. incana* (Humphries et al., 2005). Petunia AN11 efficiently rescued all traits in most T1 lines [see also (Payne et al., 2000)]. Consistent with previous results, the maize PAC1 also rescued most T1 lines (Carey et al., 2004). MP1 expression did not rescue the *ttg1* phenotypes.

**Table 1:** T1 plants or T2 lines transformed with pAMPAT-35S-WDR showing phenotypic rescue (rescued lines/total number of lines) in *ttg1.*

Gene	Trichome	Root hair	Anthocyanin	Seed coat color	Seed coat mucilage
*AtTTG1*	**25/27**	**23/27**	**22/27**	**25/27**	**25/27**
*AaTTG1*	**15/16**	**13/16**	**12/16**	**13/16**	**14/16**
*GhTTG1*	**35/50**	**33/50**	**33/50**	**35/50**	**35/50**
*GhTTG2*	0/15	0/15	0/15	0/15	0/15
*GhTTG3*	**14/19**	**12/19**	**12/19**	**13/19**	**13/19**
*GhTTG4*	0/16	0/16	0/16	0/16	0/16
*PhAN11*	**12/15**	**12/15**	**12/15**	**12/15**	**12/15**
*ZmPAC1*	**34/35**	**30/35**	**30/35**	**32/35**	**34/35**
*ZmMP1*	0/12	0/12	0/12	0/12	0/12

*Bold values indicate rescue*.

### Rescue of the *Arabidopsis gl3 egl3 tt8 Mutant by bHLH* Homologs

*bHLHs* proteins involved in MBW functions in the context of the five traits analyzed are typically important for some but not all traits. In order to study the function of bHLH homologs in Arabidopsis we used the *gl3 egl3 tt8* triple mutant in Col background. This triple mutant exhibits defects in all five traits similarly as *ttg1* mutants (Zhang et al., 2003).

In a first step, we studied the rescue ability of the Arabidopsis bHLH proteins AtGL3, AtEGL3, and AtTT8 (Table 2). The genetic analysis had revealed partially overlapping functions of these genes such that trichome and root hair formation are controlled by AtGL3, AtEGL3, anthocyanidin production by AtGL3, AtEGL3, and AtTT8, seed color by AtTT8, seed coat mucilage by AtEGL3 and AtTT8 (Zhang et al., 2003). To distinguish, whether this differential function is due to differential expression or different protein functions, we expressed the respective CDS in the *gl3 egl3 tt8* mutant. In addition, we included AtMYC1 which is involved in trichome and root hair development but has a molecular function distinct from AtGL3 and AtEGL3 and cannot rescue the *gl3 egl3* mutant trichome phenotype (Zhao et al., 2012; Pesch et al., 2013). We noted that none of the genes rescued seed coat color. We reasoned that the 35S promoter might not be sufficient for driving the expression in the seed coat. We therefore performed a second set of experiments in which the bHLH were expressed under the *TT8* promoter in *tt8* mutants and used these results for the interpretation (Table 3).

**Table 2:** T1 plants or T2 lines transformed by pAMPAT-35S:bHLH showing phenotypic rescue (rescued lines/total number of lines) in *gl3/egl3/tt8*.

Gene	Trichome	Root hair	Anthocyanin	Seed coat color	Seed coat mucilage
*AtGL3*	**18/20**	**14/20**	**18/20**	0/20	0/20
*AtEGL3*	**15/20**	**10/20**	**10/20**	0/20	**9/20**
*AtMYC1*	0/20	**7/20**	0/20	0/20	**14/20**
*AtTT8*	0/20	0/20	**13/20**	0/20	**18/20**
*AaGL3*	**25/28**	**16/28**	**24/28**	0/28	**13/28**
*AaEGL3*	**10/13**	**7/13**	**10/13**	0/13	**(6/13)^a^**
*AaMYC1*	0/10	**3/10**	0/10	0/10	**6/10**
*AaTT8*	0/20	0/20	**12/20**	0/20	**18/20**
*GhDEL61*	0/20	**12/20**	**9/20**	0/20	**17/20**
*GhDEL65*	0/20	**13/20**	**10/20**	0/20	**18/20**
*PhAN1*	0/20	0/20	**11/20**	0/20	**10/20**
*PhJAF13*	0/19	**10/19**	0/19	0/19	**18/19**
*ZmR(Lc)*	**20/24**	**13/24**	**20/24**	0/24	**(10/24)^a^**
*ZmR(S)*	**20/22**	**15/22**	**20/22**	0/22	**(4/22)^a^**
*ZmB*	0/10	0/10	0/10	0/10	0/10

^a^*Partially rescued. Bold values indicate rescue*.

**Table 3:** T1 plants or T2 lines transformed by pAMPAT-pro*TT8*:bHLH showing phenotypic rescue (rescued lines/total number of lines) in *tt8* single mutant.

*Gene*	Trichome	Seed coat color
*AtGL3*	0/20	0/20
*AtEGL3*	0/20	**(5/20)^b^**
*AtMYC1*	0/20	0/20
*AtTT8*	0/20	**18/20**
*AaGL3*	0/20	0/20
*AaEGL3*	0/20	0/20
*AaMYC1*	0/20	0/20
*AaTT8*	0/20	**15/20**
*GhDEL61*	0/20	0/20
*GhDEL65*	0/20	0/20
*PhAN1*	0/20	**12/20**
*PhJAF13*	0/20	0/20
*ZmR(Lc)*	0/20	**20/20**
*ZmR(S)*	0/20	**19/20**
*ZmB*	0/20	0/20

^b^*Seed coat is light brown. Bold values indicate rescue*.

Consistent with the genetic results, AtGL3 rescued trichome and root hair formation and anthocyanidin production but not seed color or mucilage release (Supplementary Figures S3–S5 and Tables 2, 3). AtEGL3 rescued all four traits in which it is also genetically relevant and in addition shows a weak rescue of seed coat color. Consistent with its genetic function, TT8 expression led to a rescue of seed color and mucilage release (Supplementary Figures S5, S6). Thus, the differential function of the analyzed bHLH proteins can be explained at the protein level. The MYC1 protein, could rescue root hair and seed mucilage release.

To analyze the function of putative orthologous bHLH proteins we focused on another Brassicaceae species, *A. alpina*. Here, orthologous genes can be identified by sequence similarity and the chromosomal context of the respective genes by studying synteny. We found similar rescue properties for most bHLH proteins with one notable exception (Tables 2, 3). In contrast to AtGL3, the AaGL3 protein was unable to rescue the seed mucilage phenotype. This indicates that the function of GL3 has diverged between the two species at the protein level.

In cotton, we chose to analyze GhDEL65 and GhDEL61 that are linked to fiber formation and that can functionally replace AtGL3 and AtEGL3 in Arabidopsis when expressed under the Arabidopsis *GL3* upstream sequences (Wang et al., 2013; Shangguan et al., 2016). We found rescue of the root hair, anthocyanidin and mucilage phenotype (Tables 2, 3). It is difficult to explain why trichome rescue was observed when the two cotton genes were expressed under the control of the *AtGL3* upstream sequences but not under the control of the 35S promoter in our studies. As the *AtGL3* gene requires regulatory intron sequences for the correct expression (Friede et al., 2017), it is possible that the different results can be explained by unknown differences in the temporal, spatial or levels of expression. However, our data clearly show that the two bHLH proteins from cotton can regulate root hair, anthocyanidin and mucilage release. Hence, by their protein function they are most similar to Arabidopsis EGL3 and clearly distinct from TT8.

We analyzed two bHLH genes from Petunia, *PhJAF13* and *PhAN1*. Mutations in *PhAN1* lead to defects in anthocyanidin production, seed color and seed coat cell differentiation (Spelt et al., 2000, 2002). PhJAF13 was shown to activate anthocyanidin production upon overexpression (Quattrocchio et al., 1998). Our rescue experiments revealed different properties (Tables 2, 3). While PhAN1 expression in the *gl3 egl3 tt8* mutant rescues the anthocyanidin, seed color and mucilage phenotypes, PhJAF13 overexpression resulted in a rescue of the root hair and seed coat mucilage phenotypes. Thus, the rescue ability of PhAN1 corresponds well with the mutant phenotype of the respective Petunia mutant. By contrast, PhJAF13 appears to have also the property to promote root hair formation (Supplementary Figure S3).

From Maize we selected three bHLH genes for our analysis, *ZmR(Lc)*, *ZmR(S)*, and *ZmB*. Mutations in all three genes result in severe anthocyanidin phenotypes (Dooner, 1979; Ludwig et al., 1989; Patterson et al., 1991; Stadler and Neuffer, 1953). Overexpression of both *ZmR* genes was sufficient to rescue all five traits whereas *ZmB* exhibited no rescue of any trait (Tables 2, 3).

### Rescue of the *Arabidopsis Mutants by R2R3MYB* Homologs

We focused our analysis of the rescue ability of R2R3MYB proteins on the rescue of an anthocyanidin mutant as an example for the most ancient trait and trichome mutants as an example for a more recently evolved trait (Table 4). For the analysis of the anthocyanidin phenotype we used the Arabidopsis *pap1 pap2* double mutant. *AtPAP1* and *AtPAP2* are close homologs and both involved in the regulation of anthocyanidin (Teng et al., 2005; Gonzalez et al., 2008). The trichome phenotype was assessed in Arabidopsis *gl1* mutants. The R2R3MYB proteins were expressed under the control of the 35S promoter. As expected both, *AtPAP1* and *AtPAP2*, rescued the double mutant efficiently. However, none of the other Arabidopsis R2R3MYB proteins including AtGL1, AtWER, AtTT2, and AtMYB61 were able to rescue the anthocyanidin phenotype. The *gl1* trichome phenotype was rescued only by GL1 but not by any of the other proteins (Supplementary Figure S7). This suggest that also the R2R3MYB protein have evolved trait specific properties. A similar result was obtained for the Arabis R2R3MYB proteins. Only the PAP-like protein AaPAPL revealed rescue of the anthocyanidin phenotype and only AaGL1 the *gl1* phenotype. In cotton we chose four R2R3MYB proteins. GhMYB2 is expressed in fibers and can rescue the Arabidopsis trichome phenotype (Wang et al., 2004). GhMYB25 is expressed in the seed coat and mutations in the gene lead to slower fiber growth (Machado et al., 2009). *GhRLC1* was shown to be involved in the regulation of anthocyanidin in cotton (Gao et al., 2013). In addition we added GhMYB3 which we found as a close homolog of GhMYB2. Consistent with the mutant phenotype, GhRLC1 rescued the anthocyanidin phenotype (Table 4). GhMYB2 and GhMYB3 rescued the *gl1* trichome phenotype, GhMYB25 exhibited no rescue. In Petunia we analyzed three genes. *PhAN2* and *PhAN4* are important for anthocyanidin production (Quattrocchio et al., 1993, 2006b; Spelt et al., 2002). *PhPH4* regulates the acidification of the vacuole in petals (Quattrocchio et al., 2006b). All three R2R3MYB proteins rescued the anthocyanidin phenotype but not the trichome phenotype. Finally, we studied the *ZmC1* gene from maize that was described to act as a regulator of anthocyanidin production (Sainz et al., 1997; Grotewold et al., 2000; Hernandez et al., 2004). Consistent with this function in maize we found that *ZmC1* rescued the *pap1 pap2* mutant but not the *gl1* mutant.

**Table 4:** T1 or T2 lines transformed by pAMPAT-35S-R2R3MYB showing phenotypic rescue (rescued lines/total number of lines) in *gl1* single mutant or *pap1pap2* mutant.

Gene	Trichome (*gl1*)	Anthocyanin (*pap1 pap2*)
*AtGL1*	**17/18**	0/16
*AtWER*	0/16	0/16
*AtPAP1*	0/16	**18/18**
*AtPAP2*	0/16	**13/18**
*AtTT2*	**(9/16)^c^**	0/16
*AtMYB61*	0/16	0/16
*AaGL1*	14/16	0/16
*AaWER*	0/16	0/16
*AaPAPL*	0/16	**17/18**
*AaMYB23*	0/16	0/16
*GhMYB2*	**14/16**	0/15
*GhMYB3*	**15/16**	0/15
*GhMYB25*	0/16	0/15
*GhRLC1*	0/16	**6/12**
*PhAN2*	0/16	**10/16**
*PhAN4*	0/16	**13/16**
*PhPH4*	0/16	**7/14**
*ZmC1*	0/16	**8/12**
*ZmPL*	**(6/16)^c^**	**7/12**
*ZmP1*	0/16	**9/15**

^c^*Trichomes on leaf margin. Bold values indicate rescue*.

### Inter-Species MBW Pairwise Interaction

It is generally thought that the specificity of MBW function is due to the formation of specific combinations of particular components. In this light, the differential rescue behavior of MBW proteins from different species might be explained by differences in the protein-protein interactions with the proteins in *A. thaliana*. Interactions of MBW proteins in each species were already extensively studied (Supplementary Table S3). To analyze the interactions of MBW proteins from other species with the Arabidopsis proteins we performed LUMIER assays [LUminescence-based Mammalian IntERactome (Blasche and Koegl, 2013)]. In LUMIER assays proteins of interest are expressed in human HEK293TM cells with one protein fused to the *S. aureus* protein A (ProtA) and the other with a *R. reniformis* luciferase (Rluc) followed by pulldown experiments and a quantification of the precipitated luciferase activity (Supplementary Figure S8 and Table 5).

**Table 5:** Analysis of inter-species MBW pairwise interaction by LUMIER pulldown assays.

		WDR		bHLH					MYB				
		**AtTTG1**	**AtGL3**	**AtEGL3**	**AtTT8**	**AtMYC1**	**AtGL1**	**AtWER**	**AtPAP1**	**AtPAP2**	**AtTT2**	**AtMYB61**	**w/o**
	AaTTG1		+/+	+/+	+/+	+/+	-/-	-/-	-/-	-/-	-/-	-/-	-/-
	GhTTG1		+/+	+/+	+/+	+/+	-/-	-/-	-/-	-/-	-/-	-/-	-/-
	GhTTG2		-/-	-/-	-/-	-/-	-/-	-/-	-/-	-/-	-/-	-/-	-/-
	GhTTG3		+/w	+/+	-/-	+/+	-/-	-/-	-/-	-/-	w/w	-/-	-/-
WDR	GhTTG4		-/-	-/-	-/-	-/-	-/-	-/-	-/-	-/-	-/-	-/-	-/-
	PhAN11		+/+	+/+	-/-	+/+	-/-	-/-	-/-	-/-	-/-	-/-	-/-
	ZmPAC1		+/+	+/+	+/+	+/+	-/-	-/-	-/-	-/-	-/-	-/-	-/-
	ZmMP1		-/-	-/-	-/-	w/-	-/-	-/-	-/-	-/-	-/-	-/-	-/-
	AaGL3	+/+					+/+	+/+	+/+	w/+	+/+	-/-	-/-
	AaEGL3	+/+					+/+	+/+	w/w	-/-	+/+	-/-	-/-
	AaTT8	+/+					+/+	+/+	+/+	+/+	+/+	-/w	-/-
	AaMYC1	+/+					+/+	+/+	+/+	+/+	+/+	-/-	-/-
bHLH	GhDEL61	+/+					+/+	+/+	+/+	+/w	+/+	-/-	-/-
	GhDEL65	+/+					+/+	+/+	+/+	+/+	+/+	-/-	-/-
	PhAN1	+/+					+/+	+/+	+/+	+/+	+/+	-/-	-/-
	PhJAF13	+/+					+/+	+/+	+/+	w/w	+/+	-/-	-/-
	ZmR(Lc)	+/+					+/+	+/+	+/+	+/+	+/+	-/-	-/-
	ZmR(S)	+/+					+/+	+/+	+/+	+/+	+/+	-/-	-/-
	ZmB	+/+					-/-	-/-	-/-	-/-	-/-	-/-	-/-
	AaGL1	-/-	+/+	+/+	+/+	+/+							-/-
	AaWER	-/-	+/+	+/+	+/+	+/+							-/-
	AaPAPL	-/-	+/+	+/+	+/+	+/+							-/-
	AaMYB23	-/-	+/+	+/+	+/+	+/+							-/-
	GhMYB2	-/-	+/+	+/+	+/+	+/+							-/-
	GhMYB3	-/-	+/+	+/+	+/+	+/+							-/-
	GhMYB25	-/-	-/-	-/-	-/-	-/-							-/-
MYB	GhRLC1	-/-	+/+	+/+	+/+	+/+							-/-
	PhAN2	-/-	+/+	+/+	+/+	+/+							-/-
	PhAN4	-/-	+/+	+/+	+/+	+/+							-/-
	PhPH4	-/-	+/+	+/+	+/+	+/+							-/-
	ZmC1	-/-	+/+	+/+	+/+	+/+							-/-
	ZmPL	w/w	+/+	+/+	+/+	+/+							-/-
	ZmP1	-/-	-/-	-/-	-/-	-/-							-/-
	w/o	-/-	-/-	-/-	-/-	-/-	-/-	-/-	-/-	-/-	-/-	-/-	-

*Arabidopsis thaliana [At], Arabis alpine [Aa], Gossypium hirsutum [Gh], Petunia hybrid [Ph] and Zea mays [Zm]. All proteins are single-expressed in human cells (HEK293TN) and immunoprecipitated with IgG Dynabeads (For full set of data please refer to Supplementary Tables S4, S5). w/o: Empty vector without CDS fusion. +: positive interaction (luciferase activity ≥ 2.5%). w: weak interaction (luciferase activity = 1.5% ∼ 2.5%). -: no interaction (luciferase activity < 1.5%)*.

In a first step we explored the binding behavior of TTG1 homologs to Arabidopsis bHLH proteins (Table 5). AaTTG1, GhTTG1, PhAN11, and ZmPAC1 exhibited interactions with all four Arabidopsis bHLH proteins. GhTTG3 interacted with AtGL3, AtEGL3, and AtMYC1 but not with AtTT8. GhTTG2, GhTTG4, and ZmMP1 proteins did not interact with any of the bHLH proteins. This correlates well with our finding that the latter four TTG1 homologs did not rescue *ttg1* mutants in Arabidopsis.

In a next step we tested the binding of homologous bHLH proteins to Arabidopsis TTG1 and MYB proteins (Table 5). All bHLH proteins tested showed an interaction with the Arabidopsis AtTTG1 protein. Binding of bHLH proteins from other species with the Arabidopsis R2R3MYB proteins varied. Most bHLH proteins showed interaction with GL1, WER, PAP1, PAP2, and TT2 but not with MYB61. Arabis EGL3 exhibited a differential binding behavior such that it interacts with GL1, WER, PAP1, and TT2 but not PAP2. We found no interactions of ZmB with any R2R3MYBs. This is consistent with the fact that ZmB possesses no rescue ability.

Finally, we studied the binding behavior of R2R3MYB from other species with Arabidopsis MBW proteins (Table 5). As expected, we generally found no binding to AtTTG1. Only ZmPL exhibited weak binding to AtTTG1. Except for GhMYB25 all R2R3MYB proteins interacted with all four bHLH proteins from Arabidopsis.

Taken together the analysis of the interactions of MBW proteins from other species with the Arabidopsis proteins can explain those cases in which we found no rescue of Arabidopsis mutants as the respective proteins do not interact with the relevant partners. However, the binding patterns do not explain any differential rescue of traits.

## Discussion

### Rescue With Arabidopsis MBW Proteins Reveals Trait-Specific Protein Properties

In Arabidopsis, the MBW complex regulates five different traits by involving different bHLH and R2R3MYBs in a trait-specific manner. This raises the question, whether trait specificity is due to transcriptional regulation or different properties of bHLH and R2R3MYB proteins. There are examples available suggesting that both aspects are relevant (Lai et al., 2013; Lloyd et al., 2017). To address this question, we expressed the MBW proteins under the 35S promoter in appropriate *A. thaliana* mutants and analyzed, which traits were rescued in the T1 generation. This enabled us to judge the ability of MBW proteins to function in the regulation of the different traits. Rescue experiments with *A. thaliana* bHLH proteins GL3, EGL3, and TT8 revealed that the rescue ability of the different traits matches their genetic requirements (Supplementary Table S1) (Zhang et al., 2003). This suggests that these bHLH proteins possess properties mediating trait specific regulation events. By contrast, the bHLH protein MYC1 is important for trichome development but cannot rescue the trichome phenotype of the *gl3 egl3 ttg8* mutant. This is consistent with previous data (Zhao et al., 2012) and can be explained by a different cellular localization and molecular function (Pesch et al., 2013). Similarly, we found some trait specificity for the R2R3MYB proteins. However, we found no rescue of *gl1* mutants with overexpressed WER. This is in conflict with previous findings where WER expression under the GL1 promoter showed rescue (Lee and Schiefelbein, 2001). This may be considered to show the limitations of the overexpression approach taken in this study. It may also reflect that an overexpression approach is more stringent such that only GL1 can rescue the *gl1* mutant but not WER.

### Evolutionary Comparison of MBW Functions

Because a rescue approach considering all five traits does not allow a systematic analysis of evolutionary conserved MBW proteins, we selected genes and species for which genetic or molecular data are available suggesting a functional relevance in one or several of the pathways under consideration. As a consequence, some of our experiments are repetitions and can serve as references and controls for comparison with previous results. Most WDR homologs studied here were able to complement *ttg1 A. thaliana* mutants. Only the two cotton proteins GhTTG2 and GhTTG4 and the maize MP1 protein did not show any rescue. This observation correlates with the phylogenetic classification that places MP1 and PAC1 in different clades (Carey et al., 2004). All WDR proteins including AtTTG1, AaTTG1, GhTTG1, GhTTG2, GhTTG2, and GhTTG4 are most similar to AtAN11, which belongs to MP1 clade (Carey et al., 2004; Humphries et al., 2005). The rescue ability of bHLH protein from other species correlates reasonably well with the function of those for which mutant data are available. This is true for the *A. alpina* proteins TT8, EGL3, petunia AN1 and maize R(LC) and R(S). A notable exception is the maize ZmB protein as the corresponding maize mutants show an anthocyanidin phenotype. As the ZmB protein does not interact with any of the *A. thaliana* R2R3MYB proteins, it is conceivable that ZmB cannot rescue because of this lack of interactions. We found a similar situation for the R2R3MYB proteins. Here all R2R3MYBs for which a function in the regulation of anthocyanidin was demonstrated by mutant phenotypes in the respective species specifically rescued the anthocyanidin phenotype but not the trichome phenotype. This includes the cotton proteins GhRCL1 (Gao et al., 2013), the petunia proteins PhAN2 and PhAN4 (Quattrocchio et al., 1993, 2006b; Spelt et al., 2002) and the maize protein ZmC1 (Sainz et al., 1997; Grotewold et al., 2000; Hernandez et al., 2004).

Previous experiments aiming to understand whether changes in the temporal-spatial regulation or changes in protein functions are relevant to adopt trait specific functions revealed examples for an importance of gene regulation. *GL1* and *WER* can equally well rescue the *wer* root hair phenotype when expressed under the *WER* promoter (Lee and Schiefelbein, 2001). Similarly, the *gl1* mutant phenotype can be rescued by the MYB82 protein when expressed under the GL1 promoter (Liang et al., 2014). Taken together, our data suggest that also changes in protein functions of MBW proteins are important. The specificity for the regulation of the five the different TTG1-dependent traits can be explained to a large degree by the protein behavior of the bHLH and R2R3MYB proteins. It will be interesting to see in future experiments to understand which biochemical properties of the bHLH and R2R3MYB proteins might be relevant for trait specific regulation.

## Data Availability

All datasets generated for this study are included in the manuscript and/or the Supplementary Files.

## Author Contributions

BZ performed the experiments. BZ and MH designed the experiments and wrote the manuscript.

## Conflict of Interest Statement

The authors declare that the research was conducted in the absence of any commercial or financial relationships that could be construed as a potential conflict of interest.
