# Supplementary material for: Evolutionary Analysis of MBW Function by Phenotypic Rescue in Arabidopsis thaliana
Source: Front Plant Sci. 2019 Mar 29;10:375. doi: 10.3389/fpls.2019.00375 (PMC6449874; doi:10.3389/fpls.2019.00375)
Supplement: Supplementary file 1 [file Data_Sheet_1.pdf]

Table S1: Inter-specific rescue experiments in *Arabidopsis* mutants

Gene Name	Promoter	Background	Outcome					References
			T	R	AN	PA	M	
AtTTG1	Pro*AtTTG1* / 35S	*ttg1*	y	*n.a.*	*n.a.*	y	y	(Bouyer, Geier et al., 2008)
AaTTG1	Pro*AtTTG1*	*ttg1*	y	*n.a.*	*n.a.*	*n.a.*	*n.a.*	(Chopra, Wolff et al., 2014b)
GhTTG1	35S	*ttg1*	y	y	y	y	y	(Humphries et al., 2005)
GhTTG2	35S	*ttg1*	n	n	n	n	n	(Humphries et al., 2005)
GhTTG3	35S	*ttg1*	y	y	y	y	y	(Humphries et al., 2005)
GhTTG4	35S	*ttg1*	n	n	n	n	n	(Humphries et al., 2005)
PhAN11	?	*ttg1*	y	y	y	y	y	(Payne et al., 2000)
ZmPAC1	35S	*ttg1*	y	y	y	y	y	(Carey, Strahle et al., 2004a)
AtGL3	35S	*ttg1*	y	*n.a.*	y	n	n	(Zhang, Gonzalez et al., 2003b)
	35S	*tt8*	*n.a.*	*n.a.*	y	y[1]	*n.a.*	(Zhang et al., 2003b)
	35S	*gl3 egl3*	y	*n.a.*	y	y[1]	*n.a.*	(Zhang et al., 2003b)
	Pro*AtGL3- cDNA*	*gl3 egl3*	n[2]	y	*n.a.*	*n.a.*	*n.a.*	(Friede et al., 2017b, Zhao, Wang et al., 2012a)
	Pro*AtGL3-Genomic*	*gl3 egl3*	y	y	*n.a.*	*n.a.*	*n.a.*	(Friede et al., 2017b, Morohashi, Zhao et al., 2007)
	Pro*AtGL3-Genomic*	*gl1*	y	*n.a.*	*n.a.*	*n.a.*	*n.a.*	Morohashi et al, 2007
	Pro*AtMYC1-cDNA*	*myc1*	y	y	*n.a.*	*n.a.*	*n.a.*	(Zhao et al., 2012a)
AtEGL3	35S / Pro*AtEGL3- Genomic*	*ttg1*	y	*n.a.*	y	y	y	(Zhang et al., 2003b)
	35S / Pro*AtEGL3- Genomic*	*tt8*	*n.a.*	*n.a.*	y	y	*n.a.*	(Zhang et al., 2003b)
	35S / Pro*AtEGL3- Genomic*	*gl3 egl3*	y	y	y	y	*n.a.*	(Zhang et al., 2003b)
	Pro*AtEGL3- cDNA*	*gl3 egl3*	y	y	*n.a.*	*n.a.*	*n.a.*	(Zhao et al., 2012a)
	Pro*AtMYC1- cDNA*	*myc1*	y	y	*n.a.*	*n.a.*	*n.a.*	(Zhao et al., 2012a)
AtTT8	35S	*tt8*	*n.a.*	*n.a.*	*n.a.*	y	*n.a.*	(Chen, Xuan et al., 2014)
AtMYC1	35S	*gl3 egl3*	n	*n.a.*	*n.a.*	*n.a.*	*n.a.*	(Pesch, Schultheiß et al., 2013b)
	35S / Pro*AtMYC1*	*myc1*	y	y	*n.a.*	*n.a.*	*n.a.*	(Pesch et al., 2013b, Symonds, Hatlestad et al., 2011a, Zhao et al., 2012a)
	Pro*AtGL3* / *AtEGL3*	*myc1*	y	y	*n.a.*	*n.a.*	*n.a.*	(Zhao et al., 2012a)
	Pro*AtGL3* / *AtEGL3*	*gl3 egl3*	n	n	*n.a.*	*n.a.*	*n.a.*	(Zhao et al., 2012a)

Table S1: Cont.

Gene Name	Promoter	Background	Outcome					References
			T	R	AN	PA	M	
GhDEL61	35S-*Genomic*	*gl3 egl3*	n[3]	*n.a.*	*n.a.*	*n.a.*	*n.a.*	(Shangguan et al., 2016)
	Pro*AtGL3*- *Genomic*	*gl3*	y	*n.a.*	*n.a.*	*n.a.*	*n.a.*	(Wang et al., 2013)
GhDEL65	35S-*Genomic*	*gl3 egl3*	y[4]	*n.a.*	*n.a.*	*n.a.*	*n.a.*	(Shangguan et al., 2016)
	Pro*AtGL3*- *Genomic*	*gl3*	y	*n.a.*	*n.a.*	*n.a.*	*n.a.*	(Wang et al., 2013)
ZmR(Lc)	35S	*ttg1*	y	*n.a*	y	y	*n.a.*	(Lloyd, Walbot et al., 1992)
AtGL1	35S	*ga1-3*	y	*n.a.*	*n.a.*	*n.a.*	*n.a.*	(Qi, Huang et al., 2014)
	Pro*AtGL1*	*gl1*	y	*n.a.*	*n.a.*	*n.a.*	*n.a.*	(Lee & Schiefelbein, 2001)
	Pro*AtWER*	*wer*	*n.a*	y	*n.a*	*n.a*	*n.a*	(Lee & Schiefelbein, 2001)
AtWER	Pro*AtGL1*	*gl1*	y	*n.a.*	*n.a.*	*n.a.*	*n.a.*	(Lee & Schiefelbein, 2001)
	Pro*AtWER*	*wer*	*n.a*	y	*n.a.*	*n.a.*	*n.a.*	(Lee & Schiefelbein, 2001)
AtPAP1	35S	*cop1-4*	*n.a.*	*n.a.*	y	*n.a.*	*n.a.*	(Maier, Schrader et al., 2013)
AtPAP2	35S	*cop1-4*	*n.a.*	*n.a.*	y	*n.a.*	*n.a.*	(Maier et al., 2013)
AtTT2	35S	*tt2*	*n.a.*	*n.a.*	*n.a.*	y	*n.a.*	(Baudry, Heim et al., 2004b)
AtMYB61	35S	*myb61*	*n.a.*	*n.a.*	*n.a.*	*n.a.*	y	(Romano, Dubos et al., 2012)
GhMYB2	35S	*gl1*	y	*n.a.*	*n.a.*	*n.a.*	*n.a.*	(Guan, Pang et al., 2014)
GhMYB25	35S	*gl1*	n	*n.a.*	*n.a.*	*n.a.*	*n.a.*	(Wu, Machado et al., 2006)
ZmC1	35S	*ttg1*	*n.a*	*n.a*	n	n	*n.a*	(Lloyd et al., 1992)

T: Trichome; R: Root hair; AN: Anthocyanidin; PA: Pro-Anthocyanidin; M: Seed coat mucilage

y: yes (rescued); n: no (not rescued); *n.a.*: data not available

1. Light brown seed coat color
2. In (Zhao et al., 2012a) , GL3:GL3(cDNA) slightly rescued trichome in leaf margin
3. *35S: GhDEL61* in wild-type plants also produced high-density trichomes on rosettes leaves and stems
4. Margins of the leaf, with a small and less branched trichome from 5th true leaf

Table S2: Transgenic plant lines

**Insertion (plasmid name)**	**Background**
pAMPAT-35S-GW-*AtTTG1*	*ttg1-1*
pAMPAT-35S-GW-*AaTTG1*	*ttg1-1*
pAMPAT-35S-GW-*GhTTG1*	*ttg1-1*
pAMPAT-35S-GW-*GhTTG2*	*ttg1-1*
pAMPAT-35S-GW-*GhTTG3*	*ttg1-1*
pAMPAT-35S-GW-*GhTTG4*	*ttg1-1*
pAMPAT-35S-GW-*PhAN11*	*ttg1-1*
pAMPAT-35S-GW-*ZmPAC1*	*ttg1-1*
pAMPAT-35S-GW-*ZmMP1*	*ttg1-1*
pAMPAT-35S-GW-*AtGL3*	*gl3egl3tt8*
pAMPAT-35S-GW-*AtEGL3*	*gl3egl3tt8*
pAMPAT-35S-GW-*AtTT8*	*gl3egl3tt8*
pAMPAT-35S-GW-*AtMYC1*	*gl3egl3tt8*
pAMPAT-35S-GW-*AaGL3*	*gl3egl3tt8*
pAMPAT-35S-GW-*AaEGL3*	*gl3egl3tt8*
pAMPAT-35S-GW-*AaTT8*	*gl3egl3tt8*
pAMPAT-35S-GW-*AaMYC1*	*gl3egl3tt8*
pAMPAT-35S-GW-*GhDEL61*	*gl3egl3tt8*
pAMPAT-35S-GW-*GhDEL65*	*gl3egl3tt8*
pAMPAT-35S-GW-*PhAN1*	*gl3egl3tt8*
pAMPAT-35S-GW-*PhJAF13*	*gl3egl3tt8*
pAMPAT-35S-GW-*ZmR(Lc)*	*gl3egl3tt8*
pAMPAT-35S-GW-*ZmR(S)*	*gl3egl3tt8*
pAMPAT-35S-GW-*ZmB*	*gl3egl3tt8*
pAMPAT-Pro*TT8*-GW-*AtGL3*	*tt8*
pAMPAT-Pro*TT8*-GW-*AtEGL3*	*tt8*
pAMPAT-Pro*TT8*-GW-*AtTT8*	*tt8*
pAMPAT-Pro*TT8*-GW-*AtMYC1*	*tt8*
pAMPAT-Pro*TT8*-GW-*AaGL3*	*tt8*
pAMPAT-Pro*TT8*-GW-*AaEGL3*	*tt8*
pAMPAT-Pro*TT8*-GW-*AaTT8*	*tt8*
pAMPAT-Pro*TT8*-GW-*AaMYC1*	*tt8*

Table S2: Cont.

Insertion (plasmid name)	Background
pAMPAT-Pro*TT8*-GW-*GhDEL61*	*tt8*
pAMPAT-Pro*TT8*-GW-*GhDEL65*	*tt8*
pAMPAT-Pro*TT8*-GW-*PhAN1*	*tt8*
pAMPAT-Pro*TT8*-GW-*PhJAF13*	*tt8*
pAMPAT-Pro*TT8*-GW-*ZmR(Lc)*	*tt8*
pAMPAT-Pro*TT8*-GW-*ZmR(S)*	*tt8*
pAMPAT-Pro*TT8*-GW-*ZmB*	*tt8*
pAMPAT-35S-GW-*AtGL1*	*gl1*
pAMPAT-35S-GW-*AtWER*	*gl1*
pAMPAT-35S-GW-*AtPAP1*	*gl1*
pAMPAT-35S-GW-*AtPAP2*	*gl1*
pAMPAT-35S-GW-*AtTT2*	*gl1*
pAMPAT-35S-GW-*AtMYB61*	*gl1*
pAMPAT-35S-GW-*AaGL1*	*gl1*
pAMPAT-35S-GW-*AaWER*	*gl1*
pAMPAT-35S-GW-*AaPAPL*	*gl1*
pAMPAT-35S-GW-*AaMYB23*	*gl1*
pAMPAT-35S-GW-*GhMYB2*	*gl1*
pAMPAT-35S-GW-*GhMYB3*	*gl1*
pAMPAT-35S-GW-*GhMYB25*	*gl1*
pAMPAT-35S-GW-*GhRLC1*	*gl1*
pAMPAT-35S-GW-*PhAN2*	*gl1*
pAMPAT-35S-GW-*PhAN4*	*gl1*
pAMPAT-35S-GW-*PhPH4*	*gl1*
pAMPAT-35S-GW-*ZmC1*	*gl1*
pAMPAT-35S-GW-*ZmPL*	*gl1*
pAMPAT-35S-GW-*ZmP1*	*gl1*
pAMPAT-35S-GW-*AtGL1*	*pap1pap2*
pAMPAT-35S-GW-*AtWER*	*pap1pap2*
pAMPAT-35S-GW-*AtPAP1*	*pap1pap2*
pAMPAT-35S-GW-*AtPAP2*	*pap1pap2*

Table S2: Cont.

Insertion (plasmid name)	Background
pAMPAT-35S-GW-*AtTT2*	*pap1pap2*
pAMPAT-35S-GW-*AtMYB61*	*pap1pap2*
pAMPAT-35S-GW-*AaGL1*	*pap1pap2*
pAMPAT-35S-GW-*AaWER*	*pap1pap2*
pAMPAT-35S-GW-*AaPAPL*	*pap1pap2*
pAMPAT-35S-GW-*AaMYB23*	*pap1pap2*
pAMPAT-35S-GW-*GhMYB2*	*pap1pap2*
pAMPAT-35S-GW-*GhMYB3*	*pap1pap2*
pAMPAT-35S-GW-*GhMYB25*	*pap1pap2*
pAMPAT-35S-GW-*GhRLC1*	*pap1pap2*
pAMPAT-35S-GW-*PhAN2*	*pap1pap2*
pAMPAT-35S-GW-*PhAN4*	*pap1pap2*
pAMPAT-35S-GW-*PhPH4*	*pap1pap2*
pAMPAT-35S-GW-*ZmC1*	*pap1pap2*
pAMPAT-35S-GW-*ZmPL*	*pap1pap2*
pAMPAT-35S-GW-*ZmP1*	*pap1pap2*

Table S3: Intra-species pairwise interactions of MBW components

Pairwise proteins	Outcome	Methods	References
**Arabidopsis**			
AtTTG1-AtTTG1	-	Yeast	(Baudry et al., 2004b)
AtTTG1-AtGL3	+	Yeast Co-IP	(Payne et al., 2000, Zhang et al., 2003b, Zhao, Morohashi et al., 2008)
AtTTG1-AtEGL3	+	Yeast	(Zhang et al., 2003b)
AtTTG1-AtTT8	+	Yeast	(Baudry, Caboche et al., 2006b, Baudry et al., 2004b)
AtTTG1-AtMYC1	+	Yeast LUMIER	(Pesch et al., 2013b, Symonds et al., 2011a)
AtTTG1-AtGL1	-	Yeast	(Symonds et al., 2011a)
AtTTG1-AtTT2	w	Yeast	(Baudry et al., 2006b, Baudry et al., 2004b)
AtGL3-AtGL3	+	Yeast	(Bernhardt et al., 2003, Payne et al., 2000, Zhang et al., 2003b)
AtGL3-AtEGL3	+	Yeast	(Zhang et al., 2003b)
AtGL3-AtMYC1	-	Yeast	(Zhao et al., 2012a)
AtGL3-AtGL1	+	Yeast LUMIER Co-IP	(Morohashi & Grotewold, 2009, Morohashi et al., 2007, Payne et al., 2000, Pesch et al., 2013b, Zhang et al., 2003b, Zhao et al., 2008)
AtGL3-AtWER	+	Yeast	(Bernhardt et al., 2003, Tominaga, Iwata et al., 2007, Zimmermann, Heim et al., 2004a)
AtGL3-AtPAP1	+	Yeast	(Baudry et al., 2006b, Zhang et al., 2003b)
AtGL3-AtPAP2	+	Yeast	(Zhang et al., 2003b)
AtGL3-AtTT2	+	Yeast	(Baudry et al., 2006b)
AtEGL3-AtEGL3	+	Yeast	(Bernhardt et al., 2003, Zhang et al., 2003b)
AtEGL3-AtGL1	+	Yeast	(Zhang et al., 2003b, Zimmermann et al., 2004a)
AtEGL3-AtWER	+	Yeast	(Tominaga et al., 2007, Zimmermann et al., 2004a)
AtEGL3-AtPAP1	+	Yeast	(Baudry et al., 2006b, Zimmermann et al., 2004a)
AtEGL3-AtPAP2	+	Yeast	(Zhang et al., 2003b, Zimmermann et al., 2004a)
AtEGL3-AtTT2	+	Yeast	(Baudry et al., 2006b, Zimmermann et al., 2004a)
AtTT8-AtTT8	+	Yeast	(Baudry et al., 2004b)
AtTT8-AtGL1	+	Yeast	(Zimmermann et al., 2004a)
AtTT8-AtWER	+	Yeast	(Zimmermann et al., 2004a)
AtTT8-AtPAP1	+	Yeast	(Zimmermann et al., 2004a)
AtTT8-AtPAP2	+	Yeast	(Zimmermann et al., 2004a)
AtTT8-AtTT2	+	Yeast	(Baudry et al., 2006b, Baudry et al., 2004b, Zimmermann et al., 2004a)

AtMYC1-AtMYC1	-	Yeast	(Zhao et al., 2012a)

Table S3: Cont.

Pairwise proteins	Outcome	Methods	References
AtMYC1-AtGL1	+	Yeast LUMIER BiFC	(Pesch et al., 2013b, Zhao et al., 2012a, Zimmermann et al., 2004a)
AtMYC1-AtWER	+	Yeast BiFC	(Zhao et al., 2012a, Zimmermann et al., 2004a)
AtMYC1-AtPAP1	+	Yeast	(Zimmermann et al., 2004a)
AtMYC1-AtPAP1	+	Yeast	(Zimmermann et al., 2004a)
AtMYC1-AtPAP1	+	Yeast	(Zimmermann et al., 2004a)
AtGL1-AtGL1	+	Yeast	(Liang, He et al., 2014)
AtPAP1-AtTT2	-	Yeast	(Baudry et al., 2006b)
AtTT2-AtTT2	+	Yeast	(Baudry et al., 2004b)
**Arabis**			
AaTTG1- AaWER	-	Yeast	(Chopra, 2015)
**Cotton**			
GhTTG3-GhDEL61	+	Yeast BiFC	(Shangguan et al., 2016)
GhTTG3-GhDEL65	+	Yeast BiFC	(Shangguan et al., 2016)
GhDEL61-GhDEL61	+	Yeast BiFC	(Shangguan et al., 2016)
GhDEL61-GhDEL65	+	Yeast BiFC	(Shangguan et al., 2016)
GhDEL61-GhMYB2	+	Yeast BiFC	(Shangguan et al., 2016, Wan, Zhang et al., 2014)
GhDEL65-GhTTG1	+	Yeast Co-IP	(Wang et al., 2013)
GhDEL65-GhDEL65	+	Yeast BiFC	(Shangguan et al., 2016)
GhDEL65-GhMYB2	+	Yeast BiFC	(Shangguan et al., 2016, Wan et al., 2014)
GhDEL65-GhMYB3	+	Yeast BiFC	(Shangguan et al., 2016)
GhMYB2-GhMYB3	-	Yeast BiFC	(Shangguan et al., 2016)
**Petunia**			
PhAN11-PhAN11	-	Yeast	(Albert, Davies et al., 2014)
PhAN11-PhAN1	+	Yeast	(Albert et al., 2014)
PhAN11-PhJAF13	+	Yeast	(Albert et al., 2014)
PhAN11-PhAN2	-	Yeast	(Albert et al., 2014)
PhAN1-PhAN1	-	Yeast Pulldown	(Albert et al., 2014, Spelt et al., 2002)
PhAN1-PhJAF13	+	Yeast	(Albert et al., 2014, Quattrocchio et al., 2006b, Spelt et al., 2002)

PhAN1-PhAN2	+	Yeast	(Albert et al., 2014)

Table S3: Cont.

Pairwise proteins	Outcome	Methods	References
PhAN1-PhPH4	+	Yeast ISH CoIP	(Quattrocchio et al., 2006b)
PhJAF13-PhJAF13	-	Yeast	(Albert et al., 2014, Quattrocchio et al., 2006b, Spelt et al., 2002)
PhJAF13-PhAN2	+	Yeast	(Albert et al., 2014)
PhJAF13-PhPH4	+	Yeast	(Quattrocchio et al., 2006b)
**Maize**			
ZmR(S)-ZmR(S)	+	Yeast Pulldown	(Feller et al., 2006, Kong, Pattanaik et al., 2012)
ZmR(S)-ZmC1	+	Yeast	(Zhang et al., 2003b, Zimmermann et al., 2004a)
ZmR(S)-ZmPL	+	Yeast	(Grotewold et al., 2000, Hernandez et al., 2004)
**Pairwise proteins**			
ZmR(S)-ZmP1	-	Yeast	(Grotewold et al., 2000, Hernandez et al., 2004)
ZmB-ZmC1	+	Yeast	(Goff, Cone et al., 1992, Hernandez et al., 2004)
ZmB-ZmPL	+	Yeast	(Goff et al., 1992)

+ : Positive interaction; w : Weak interaction; - : No interaction

Yeast: Yeast two hybrid; Co-IP: Co-immunoprecipitation; BiFC: Bimolecular Fluorescence Complement; ISH: In situ hybridization

Table S4: Interaction between TTG1, bHLHs and R2R3MYBs homologs from *Arabidopsis*

**ProtA fusion**	**Luciferase fusion**	**Luciferase activity: pulldown/input ratio (%)**	
AaTTG1	AtGL3	36.35±2.64	+
GhTTG1	AtGL3	27.38±2.23	+
GhTTG2	AtGL3	0.57±0.16	-
GhTTG3	AtGL3	2.44±0.34	w
GhTTG4	AtGL3	0.74±0.03	-
PhAN11	AtGL3	17.18±1.64	+
ZmPAC1	AtGL3	26.44±8.70	+
ZmMP1	AtGL3	0.77±0.00	-
w/o	AtGL3	0.58±0.06	-
AaTTG1	AtEGL3	20.86±9.54	+
GhTTG1	AtEGL3	25.92±1.71	+
GhTTG2	AtEGL3	0.58±0.14	-
GhTTG3	AtEGL3	6.16±4.64	+
GhTTG4	AtEGL3	0.54±0.14	-
PhAN11	AtEGL3	23.90±2.75	+
ZmPAC1	AtEGL3	28.14±3.96	+
ZmMP1	AtEGL3	0.50±0.09	-
w/o	AtEGL3	0.55±0.07	-
AaTTG1	AtTT8	23.15±3.19	+
GhTTG1	AtTT8	17.83±4.90	+
GhTTG2	AtTT8	0.59±0.07	-
GhTTG3	AtTT8	1.21±0.15	-
GhTTG4	AtTT8	0.60±0.10	-
PhAN11	AtTT8	20.55±4.53	+
ZmPAC1	AtTT8	11.51±1.23	+
ZmMP1	AtTT8	1.05±0.17	-
w/o	AtTT8	0.59±0.06	-
AaTTG1	AtMYC1	41.010.08	+
GhTTG1	AtMYC1	33.26±0.09	+

Table S4: Cont.

ProtA fusion	Luciferase fusion	Luciferase activity: pulldown/input ratio (%)	
GhTTG2	AtMYC1	0.69±0.12	-
GhTTG3	AtMYC1	20.83±0.11	+
GhTTG4	AtMYC1	0.71±0.07	-
PhAN11	AtMYC1	13.79±2.04	+
ZmPAC1	AtMYC1	45.63±3.67	+
ZmMP1	AtMYC1	1.69±0.21	w
w/o	AtMYC1	0.56±0.06	-
AaTTG1	AtGL1	0.63±0.056	-
GhTTG1	AtGL1	0.66±0.03	-
GhTTG2	AtGL1	0.67±0.01	-
GhTTG3	AtGL1	0.65±0.01	-
GhTTG4	AtGL1	0.60±0.09	-
PhAN11	AtGL1	0.61±0.01	-
ZmPAC1	AtGL1	0.65±0.10	-
ZmMP1	AtGL1	0.60±0.05	-
w/o	AtGL1	0.57±0.07	-
AaTTG1	AtWER	0.59±0.06	-
GhTTG1	AtWER	0.63±0.07	-
GhTTG2	AtWER	0.60±0.06	-
GhTTG3	AtWER	0.61±0.04	-
GhTTG4	AtWER	0.67±0.06	-
PhAN11	AtWER	0.59±0.08	-
ZmPAC1	AtWER	0.69±0.09	-
ZmMP1	AtWER	0.64±0.01	-
w/o	AtWER	0.58±0.06	-
AaTTG1	AtMYB61	0.62±0.02	-
GhTTG1	AtMYB61	0.60±0.01	-
GhTTG2	AtMYB61	0.62±0.01	-
GhTTG3	AtMYB61	0.68±0.10	-
GhTTG4	AtMYB61	0.65±0.07	-

Table S4: Cont.

ProtA fusion	Luciferase fusion	Luciferase activity: pulldown/input ratio (%)	
PhAN11	AtMYB61	0.60±0.03	-
ZmPAC1	AtMYB61	0.63±0.01	-
ZmMP1	AtMYB61	0.62±0.02	-
w/o	AtMYB61	0.57±0.01	-
AaTTG1	AtTT2	0.71±0.07	-
GhTTG1	AtTT2	1.13±0.11	-
GhTTG2	AtTT2	0.68±0.15	-
GhTTG3	AtTT2	2.02±0.21	w
GhTTG4	AtTT2	0.69±0.05	-
PhAN11	AtTT2	0.88±0.17	-
ZmPAC1	AtTT2	0.93±0.20	-
ZmMP1	AtTT2	0.69±0.02	-
w/o	AtTT2	0.60±0.07	-
AaTTG1	AtPAP1	0.71±0.07	-
GhTTG1	AtPAP1	1.13±0.11	-
GhTTG2	AtPAP1	0.68±0.15	-
GhTTG3	AtPAP1	1.02±0.21	-
GhTTG4	AtPAP1	0.69±0.05	-
PhAN11	AtPAP1	0.88±0.17	-
ZmPAC1	AtPAP1	0.93±0.20	-
ZmMP1	AtPAP1	0.69±0.02	-
w/o	AtPAP1	0.60±0.07	-
AaTTG1	AtPAP2	0.66±0.04	-
GhTTG1	AtPAP2	0.80±0.08	-
GhTTG2	AtPAP2	0.69±0.05	-
GhTTG3	AtPAP2	0.72±0.11	-
GhTTG4	AtPAP2	0.62±0.07	-
PhAN11	AtPAP2	0.88±0.17	-
ZmPAC1	AtPAP2	0.93±0.20	-
ZmMP1	AtPAP2	0.69±0.02	-

Table S4 Cont.

ProtA fusion	Luciferase fusion	Luciferase activity: pulldown/input ratio (%)	
w/o	AtPAP2	0.60±0.07	-
AaTTG1	w/o	0.56±0.08	-
GhTTG1	w/o	0.64±0.09	-
GhTTG2	w/o	0.66±0.03	-
GhTTG3	w/o	0.59±0.02	-
GhTTG4	w/o	0.69±0.10	-
PhAN11	w/o	0.67±0.05	-
ZmPAC1	w/o	0.69±0.06	-
ZmMP1	w/o	0.55±0.11	-
w/o	w/o	0.54±0.01	-
AtGL3	AaTTG1	23.29±8.03	+
AtGL3	GhTTG1	20.11±7.74	+
AtGL3	GhTTG2	0.64±0.05	-
AtGL3	GhTTG3	26.89±6.41	+
AtGL3	GhTTG4	0.72±0.13	-
AtGL3	PhAN11	15.71±6.73	+
AtGL3	ZmPAC1	26.61±4.19	+
AtGL3	ZmMP1	0.70±0.03	-
AtGL3	w/o	0.59±0.04	-
AtEGL3	AaTTG1	27.06±3.13	+
AtEGL3	GhTTG1	26.52±9.50	+
AtEGL3	GhTTG2	0.71±0.06	-
AtEGL3	GhTTG3	11.38±2.80	+
AtEGL3	GhTTG4	0.66±0.13	-
AtEGL3	PhAN11	18.03±9.12	+
AtEGL3	ZmPAC1	12.55±7.87	+
AtEGL3	ZmMP1	0.66±0.05	-
AtEGL3	w/o	0.59±0.03	-
AtTT8	AaTTG1	23.34±4.25	+
AtTT8	GhTTG1	26.57±6.30	+

Table S4: Cont.

ProtA fusion	Luciferase fusion	Luciferase activity: pulldown/input ratio (%)	
AtTT8	GhTTG2	0.81±0.13	-
AtTT8	GhTTG3	0.92±0.23	-
AtTT8	GhTTG4	0.67±0.11	-
AtTT8	ZmAN11	8.31±1.41	+
AtTT8	ZmPAC1	20.50±4.54	+
AtTT8	ZmMP1	0.70±0.11	-
AtTT8	w/o	0.59±0.08	-
AtMYC1	AaTTG1	17.22±8.33	+
AtMYC1	GhTTG1	26.87±7.34	-
AtMYC1	GhTTG2	0.69±0.02	+
AtMYC1	GhTTG3	23.63±5.74	-
AtMYC1	GhTTG4	0.67±0.21	+
AtMYC1	PhAN11	30.54±0.38	+
AtMYC1	ZmPAC1	9.17±1.17	+
AtMYC1	ZmMP1	0.70±0.40	-
AtMYC1	w/o	0.55±0.01	+
AtGL1	AaTTG1	0.67±0.08	-
AtGL1	GhTTG1	0.66±0.02	-
AtGL1	GhTTG2	0.69±0.06	-
AtGL1	GhTTG3	0.68±0.14	-
AtGL1	GhTTG4	0.71±0.09	-
AtGL1	PhAN11	0.65±0.06	-
AtGL1	ZmPAC1	0.68±0.10	-
AtGL1	ZmMP1	0.68±0.08	-
AtGL1	w/o	0.59±0.03	-
AtWER	AaTTG1	0.67±0.08	-
AtWER	GhTTG1	0.66±0.02	-
AtWER	GhTTG2	0.69±0.06	-
AtWER	GhTTG3	0.68±0.14	-
AtWER	GhTTG4	0.71±0.09	-
AtWER	PhAN11	0.65±0.06	-

Table S4: Cont.

ProtA fusion	Luciferase fusion	Luciferase activity: pulldown/input ratio (%)	
AtWER	ZmPAC1	0.68±0.10	-
AtWER	ZmMP1	0.68±0.08	-
AtWER	w/o	0.59±0.03	-
AtMYB61	AaTTG1	0.67±0.08	-
AtMYB61	GhTTG1	0.66±0.02	-
AtMYB61	GhTTG2	0.69±0.06	-
AtMYB61	GhTTG3	0.68±0.14	-
AtMYB61	GhTTG4	0.71±0.09	-
AtMYB61	PhAN11	0.65±0.06	-
AtMYB61	ZmPAC1	0.68±0.10	-
AtMYB61	ZmMP1	0.68±0.08	-
AtMYB61	w/o	0.59±0.03	-
AtTT2	AaTTG1	0.82±0.10	-
AtTT2	GhTTG1	0.86±0.07	-
AtTT2	GhTTG2	0.79±0.06	-
AtTT2	GhTTG3	1.88±0.19	w
AtTT2	GhTTG4	0.66±0.09	-
AtTT2	PhAN11	0.62±0.03	-
AtTT2	ZmPAC1	0.64±0.05	-
AtTT3	ZmMP1	0.69±0.02	-
AtTT4	w/o	0.58±0.00	-
AtPAP1	AaTTG1	0.70±0.05	-
AtPAP1	GhTTG1	0.68±0.01	-
AtPAP1	GhTTG2	0.66±0.02	-
AtPAP1	GhTTG3	0.66±0.04	-
AtPAP1	GhTTG4	0.69±0.03	-
AtPAP1	PhAN11	0.63±0.09	-
AtPAP1	ZmPAC1	0.68±0.02	-
AtPAP1	ZmMP1	0.64±0.03	-
AtPAP1	w/o	0.59±0.01	-

Table S4: Cont.

**ProtA fusion**	**Luciferase fusion**	**Luciferase activity: pulldown/input ratio (%)**	
AtPAP2	AaTTG1	0.63±0.03	-
AtPAP2	GhTTG1	0.65±0.04	-
AtPAP2	GhTTG2	0.61±0.01	-
AtPAP2	GhTTG3	0.60±0.06	-
AtPAP2	GhTTG4	0.62±0.03	-
AtPAP2	PhAN11	0.63±0.00	-
AtPAP2	ZmPAC1	0.67±0.08	-
AtPAP2	ZmMP1	0.63±0.06	-
AtPAP2	w/o	0.58±0.07	-
w/o	AaTTG1	0.67±0.06	-
w/o	GhTTG1	0.63±0.00	-
w/o	GhTTG2	0.68±0.08	-
w/o	GhTTG3	0.63±0.01	-
w/o	GhTTG4	0.62±0.00	-
w/o	PhAN11	0.69±0.11	-
w/o	ZmPAC1	0.70±0.08	-
w/o	ZmMP1	0.62±0.00	-
w/o	w/o	0.53±0.02	-

Data are mean ± s.d. (n = 3).

w/o: Empty vector without CDS fusion.

+ : Positive interaction (Luciferase activity ≥ 2.5%)

w : Weak interaction (Luciferase activity = 1.5% ~ 2.5%)

- : No interaction (Luciferase activity < 1.5%)

Table S5: Interaction between bHLH homologs and AtTTG1/R2R3MYBs from *Arabidopsis*

ProtA fusion	Luciferase fusion	Luciferase activity: pulldown/input ratio (%)	
AtTTG1	AaGL3	27.06±2.07	+
AtTTG1	AaEGL3	14.60±1.96	+
AtTTG1	AaMYC1	18.25±1.45	+
AtTTG1	AaTT8	17.93±1.43	+
AtTTG1	GhDEL61	8.84±1.78	+
AtTTG1	GhDEL65	18.86±2.26	+
AtTTG1	PhJAF13	29.06±4.13	+
AtTTG1	PhAN1	19.27±1.78	+
AtTTG1	ZmR(Lc)	32.02±3.92	+
AtTTG1	ZmR(S)	28.75±3.24	+
AtTTG1	ZmB	26.26±3.56	+
AtTTG1	w/o	0.60±0.01	-
AtGL1	AaGL3	14.17±2.36	+
AtGL1	AaEGL3	6.63±1.02	+
AtGL1	AaMYC1	4.25±0.03	+
AtGL1	AaTT8	7.85±0.41	+
AtGL1	GhDEL61	12.75±0.79	+
AtGL1	GhDEL65	8.66±0.10	+
AtGL1	PhJAF13	12.76±2.61	+
AtGL1	PhAN1	6.55±0.66	+
AtGL1	ZmR(Lc)	32.06±3.16	+
AtGL1	ZmR(S)	31.36±2.35	+
AtGL1	ZmB	0.95±0.01	-
AtGL1	w/o	0.59±0.00	-
AtWER	AaGL3	37.17±2.56	+
AtWER	AaEGL3	16.09±1.58	+
AtWER	AaMYC1	8.84±0.79	+
AtWER	AaTT8	7.67±0.87	+
AtWER	GhDEL61	20.60±0.21	+
AtWER	GhDEL65	8.61±0.24	+
AtWER	PhJAF13	30.55±2.63	+

Table S5 Cont.

ProtA fusion	Luciferase fusion	Luciferase activity: pulldown/input ratio (%)	
AtWER	PhAN1	22.92±3.75	+
AtWER	ZmR(Lc)	32.48±5.25	+
AtWER	ZmR(S)	38.22±3.55	+
AtWER	ZmB	0.90±0.02	-
AtWER	w/o	0.60±0.00	-
AtMYB61	AaGL3	0.98±0.02	-
AtMYB61	AaEGL3	0.89±0.01	-
AtMYB61	AaMYC1	0.86±0.00	-
AtMYB61	AaTT8	1.22±0.03	-
AtMYB61	GhDEL61	1.17±0.03	-
AtMYB61	GhDEL65	1.11±0.02	-
AtMYB61	PhJAF13	0.96±0.02	-
AtMYB61	PhAN1	1.36±0.03	-
AtMYB61	ZmR(Lc)	1.08±0.04	-
AtMYB61	ZmR(S)	0.93±0.02	-
AtMYB61	ZmB	0.76±0.04	-
AtMYB61	w/o	0.60±0.03	-
AtTT2	AaGL3	16.19±2.87	+
AtTT2	AaEGL3	8.58±0.41	+
AtTT2	AaMYC1	9.18±1.37	+
AtTT2	AaTT8	41.91±3.91	+
AtTT2	GhDEL61	15.37±2.25	+
AtTT2	GhDEL65	7.96±0.03	+
AtTT2	PhJAF13	24.48±1.17	+
AtTT2	PhAN1	40.54±7.54	+
AtTT2	ZmR(Lc)	22.88±2.56	+
AtTT2	ZmR(S)	25.48±3.02	+
AtTT2	ZmB	0.88±0.04	-
AtTT2	w/o	0.60±0.01	-

Table S5 Cont.

ProtA fusion	Luciferase fusion	Luciferase activity: pulldown/input ratio (%)	
AtPAP1	AaGL3	5.63±0.52	+
AtPAP1	AaEGL3	2.18±0.41	w
AtPAP1	AaMYC1	5.90±0.62	+
AtPAP1	AaTT8	6.91±1.10	+
AtPAP1	GhDEL61	6.71±0.26	+
AtPAP1	GhDEL65	5.96±1.10	+
AtPAP1	PhJAF13	8.45±1.12	+
AtPAP1	PhAN1	6.27±0.78	+
AtPAP1	ZmR(Lc)	8.21±0.67	+
AtPAP1	ZmR(S)	6.88±0.13	+
AtPAP1	ZmB	0.89±0.01	-
AtPAP1	w/o	0.60±0.00	-
AtPAP2	AaGL3	2.28±0.12	w
AtPAP2	AaEGL3	1.09±0.14	-
AtPAP2	AaMYC1	1.99±0.10	w
AtPAP2	AaTT8	3.11±0.16	+
AtPAP2	GhDEL61	8.01±0.09	+
AtPAP2	GhDEL65	5.59±0.17	+
AtPAP2	PhJAF13	2.15±0.46	w
AtPAP2	PhAN1	4.12±0.23	+
AtPAP2	ZmR(Lc)	14.42±0.77	+
AtPAP2	ZmR(S)	16.68±2.11	+
AtPAP2	ZmB	0.86±0.02	-
AtPAP2	w/o	0.60±0.03	-
w/o	AaGL3	0.72±0.01	-
w/o	AaEGL3	0.73±0.01	-
w/o	AaMYC1	0.77±0.00	-
w/o	AaTT8	0.72±0.01	-
w/o	GhDEL61	0.70±0.00	-
w/o	GhDEL65	0.73±0.02	-
w/o	PhJAF13	0.73±0.01	-

Table S5: Cont.

ProtA fusion	Luciferase fusion	Luciferase activity: pulldown/input ratio (%)	
w/o	PhAN1	0.71±0.00	-
w/o	ZmR(Lc)	0.72±0.01	-
w/o	ZmR(S)	0.69±0.00	-
w/o	ZmB	0.69±0.02	-
w/o	w/o	0.60±0.01	-
AaGL3	AtTTG1	30.58±6.14	+
AaEGL3	AtTTG1	27.55±3.65	+
AaMYC1	AtTTG1	14.13±1.22	+
AaTT8	AtTTG1	16.11±1.36	+
GhDEL61	AtTTG1	5.86±0.84	+
GhDEL65	AtTTG1	21.17±3.16	+
PhJAF13	AtTTG1	10.44±2.16	+
PhAN1	AtTTG1	16.28±2.14	+
ZmR(Lc)	AtTTG1	16.01±3.91	+
ZmR(S)	AtTTG1	7.87±2.15	+
ZmB	AtTTG1	18.31±3.10	+
w/o	AtTTG1	0.57±0.03	-
AaGL3	AtGL1	21.46±4.37	+
AaEGL3	AtGL1	21.65±3.14	+
AaMYC1	AtGL1	23.28±2.35	+
AaTT8	AtGL1	6.16±0.39	+
GhDEL61	AtGL1	10.85±1.84	+
GhDEL65	AtGL1	10.79±2.09	+
PhJAF13	AtGL1	10.25±1.07	+
PhAN1	AtGL1	10.74±2.19	+
ZmR(Lc)	AtGL1	23.35±3.09	+
ZmR(S)	AtGL1	16.70±1.70	+
ZmB	AtGL1	0.77±0.04	-
w/o	AtGL1	0.62±0.02	-
AaGL3	AtWER	23.62±3.88	+

Table S5: Cont.

ProtA fusion	Luciferase fusion	Luciferase activity: pulldown/input ratio (%)	
AaEGL3	AtWER	20.87±1.27	+
AaMYC1	AtWER	15.28±1.82	+
AaTT8	AtWER	6.78±0.45	+
GhDEL61	AtWER	11.82±1.51	+
GhDEL65	AtWER	11.62±0.85	+
PhJAF13	AtWER	6.96±0.73	+
PhAN1	AtWER	14.55±2.19	+
ZmR(Lc)	AtWER	20.82±2.88	+
ZmR(S)	AtWER	20.08±1.08	+
ZmB	AtWER	0.86±0.05	-
w/o	AtWER	0.61±0.00	-
AaGL3	AtMYB61	0.91±0.11	-
AaEGL3	AtMYB61	0.89±0.00	-
AaMYC1	AtMYB61	0.87±0.03	-
AaTT8	AtMYB61	1.74±0.21	w
GhDEL61	AtMYB61	0.90±0.01	-
GhDEL65	AtMYB61	0.93±0.03	-
PhJAF13	AtMYB61	0.94±0.01	-
PhAN1	AtMYB61	0.86±0.02	-
ZmR(Lc)	AtMYB61	0.93±0.03	-
ZmR(S)	AtMYB61	0.91±0.01	-
ZmB	AtMYB61	0.78±0.04	-
w/o	AtMYB61	0.67±0.03	-
AaGL3	AtTT2	5.72±0.28	+
AaEGL3	AtTT2	6.60±0.89	+
AaMYC1	AtTT2	9.46±0.50	+
AaTT8	AtTT2	12.54±1.21	+
GhDEL61	AtTT2	13.56±1.89	+
GhDEL65	AtTT2	13.99±0.50	+
PhJAF13	AtTT2	8.68±0.97	+
PhAN1	AtTT2	14.25±0.94	+

Table S5: Cont.

ProtA fusion	Luciferase fusion	Luciferase activity: pulldown/input ratio (%)	
ZmR(Lc)	AtTT2	17.23±1.06	+
ZmR(S)	AtTT2	16.74±1.73	+
ZmB	AtTT2	0.83±0.02	-
w/o	AtTT2	0.55±0.01	-
AaGL3	AtPAP1	5.32±0.90	+
AaEGL3	AtPAP1	1.95±0.28	w
AaMYC1	AtPAP1	3.24±0.48	+
AaTT8	AtPAP1	8.13±0.85	+
GhDEL61	AtPAP1	4.16±0.12	+
GhDEL65	AtPAP1	4.02±0.26	+
PhJAF13	AtPAP1	3.82±0.21	+
PhAN1	AtPAP1	2.96±0.14	+
ZmR(Lc)	AtPAP1	8.50±0.69	+
ZmR(S)	AtPAP1	9.38±0.93	+
ZmB	AtPAP1	0.72±0.06	-
w/o	AtPAP1	0.56±0.01	-
AaGL3	AtPAP2	3.68±0.18	+
AaEGL3	AtPAP2	1.09±0.21	-
AaMYC1	AtPAP2	5.41±0.17	+
AaTT8	AtPAP2	13.90±0.49	+
GhDEL61	AtPAP2	2.12±0.50	w
GhDEL65	AtPAP2	5.67±1.36	+
PhJAF13	AtPAP2	2.10±0.32	w
PhAN1	AtPAP2	6.45±0.35	+
ZmR(Lc)	AtPAP2	10.31±2.02	+
ZmR(S)	AtPAP2	15.15±1.15	+
ZmB	AtPAP2	1.06±0.30	-
w/o	AtPAP2	0.55±0.00	-
AaGL3	w/o	0.60±0.03	-
AaEGL3	w/o	0.58±0.00	-

Table S5: Cont.

ProtA fusion	Luciferase fusion	Luciferase activity: pulldown/input ratio (%)	
AaMYC1	w/o	0.64±0.01	-
AaTT8	w/o	0.60±0.03	-
GhDEL61	w/o	0.60±0.04	-
GhDEL65	w/o	0.58±0.03	-
PhJAF13	w/o	0.61±0.03	-
PhAN1	w/o	0.58±0.01	-
ZmR(Lc)	w/o	0.62±0.01	-
ZmR(S)	w/o	0.64±0.02	-
ZmB	w/o	0.65±0.03	-
w/o	w/o	0.55±0.01	-

Data are mean ± s.d. (n = 3).

w/o: Empty vector without CDS fusion.

+ : Positive interaction (Luciferase activity ≥ 2.5%)

w : Weak interaction (Luciferase activity = 1.5% ~ 2.5%)

- : No interaction (Luciferase activity < 1.5%)
