# Supplementary material for: Evolutionary Analysis of MBW Function by Phenotypic Rescue in Arabidopsis thaliana
Source: Front Plant Sci. 2019 Mar 29;10:375. doi: 10.3389/fpls.2019.00375 (PMC6449874; doi:10.3389/fpls.2019.00375)
Supplement: Supplementary file 2 [file Data_Sheet_2.pdf]

Figure S1

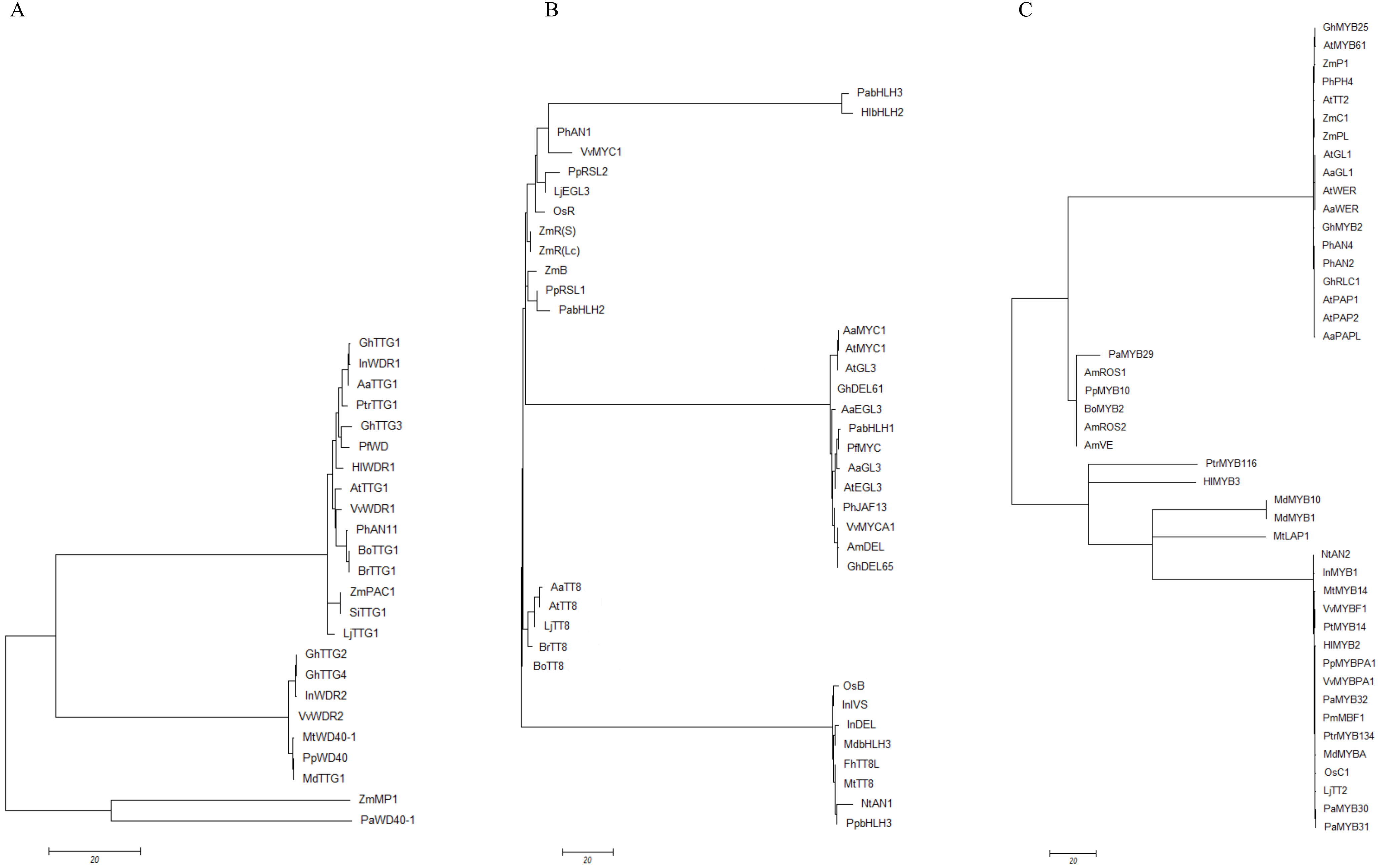
A
GhTTG1
InWDR1
AaTTG1
PtrTTG1
GhTTG3
PfWD
HlWDR1
AtTTG1
VvWDR1
PhAN11
BoTTG1
BrTTG1
ZmPAC1
SiTTG1
LjTTG1
GhTTG2
GhTTG4
InWDR2
VvWDR2
MtWD40-1
PpWD40
MdTTG1
ZmMP1
PaWD40-1
20
B
PabHLH3
HlbHLH2
PhAN1
VvMYC1
PpRSL2
LjEGL3
OsR
ZmR(S)
ZmR(Lc)
ZmB
PpRSL1
PabHLH2
AaMYC1
AtMYC1
AtGL3
GhDEL61
AaEGL3
PabHLH1
PfMYC
AaGL3
AtEGL3
PhJAF13
VvMYCA1
AmDEL
GhDEL65
AaTT8
AtTT8
LjTT8
BrTT8
BoTT8
OsB
InIVS
InDEL
MdbHLH3
FhTT8L
MtTT8
NtAN1
PpbHLH3
20
C
GhMYB25
AtMYB61
ZmP1
PhPH4
AtTT2
ZmC1
ZmPL
AtGL1
AaGL1
AtWER
AaWER
GhMYB2
PhAN4
PhAN2
GhRLC1
AtPAP1
AtPAP2
AaPAPL
PaMYB29
AmROS1
PpMYB10
BoMYB2
AmROS2
AmVE
PtrMYB116
HlMYB3
MdMYB10
MdMYB1
MtLAP1
NtAN2
InMYB1
MtMYB14
VvMYBF1
PtMYB14
HlMYB2
PpMYBPA1
VvMYBPA1
PaMYB32
PmMBF1
PtrMYB134
MdMYBA
OsC1
LjTT2
PaMYB30
PaMYB31
20

Figure S1: Phylogenetic trees of WDR, bHLH and R2R3MYB proteins

Unrooted Maximum Likelihood Phylogenetic Trees for three groups of transcriptional regulators, WDR proteins (A), bHLH transcription regulators (B) and R2R3MYB transcription factors (C). Evolutionary analyses were conducted in MEGA6 (Tamura, Stecher et al. 2013) using entire amino acid sequences based on the JTT matrix-based model (Jones, Taylor et al. 1992). The scale shows the 20 amino acids substitution per site. The abbreviation shown in front of each protein indicates the plant species: In, *Ipomoea nil*; At, *Arabidopsis thaliana*; Aa, *Arabis alpina*; Am, *Antirrhinum majus*; Bo, *Brassica oleracea*; Br, *Brassica rapa*; Gh, *Gossypium hirsutum*; Pa, *Picea abies*; Pf, *Perilla frutescens*; Ph, *Petunia hybrida*; Vv, *Vitis vinifera*; Zm, *Zea mays*. Nt, *Nicotiana tabacum*; Os, *Oryza sativa*; Pm, *Picea mariana*; Pp, *Physcomitrella patens*; Pp, *Prunus persica*; Pt, *Pinus taeda*; Ptr, *Populus trichocarpa*; Si, *Setaria italica*; In, *Ipomoea nil*; Lj, *Lotus japonicus*; Md, *Malus domestica*; Mt, *Medicago truncatula*; Hl, *Humulus lupulus*; Fh, *Freesia hybrida*.

Figure S2

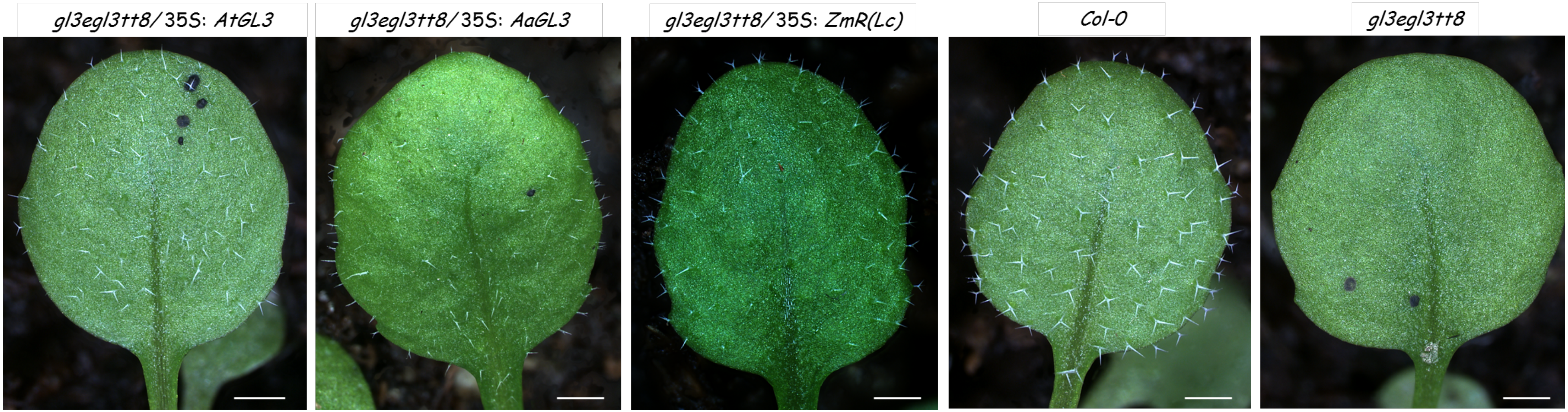

Figure S2. Third rosette leaf trichome phenotype of the *gl3egl3tt8* triple mutant rescued with 35S:*bHLHs*. Leaves of 10 days old plants are shown. Typically, 35S-overexpression of bHLH proteins result in a rescue of trichomes with less regular patterns compared to wild type. (Scale bar =5 mm).

Figure S3

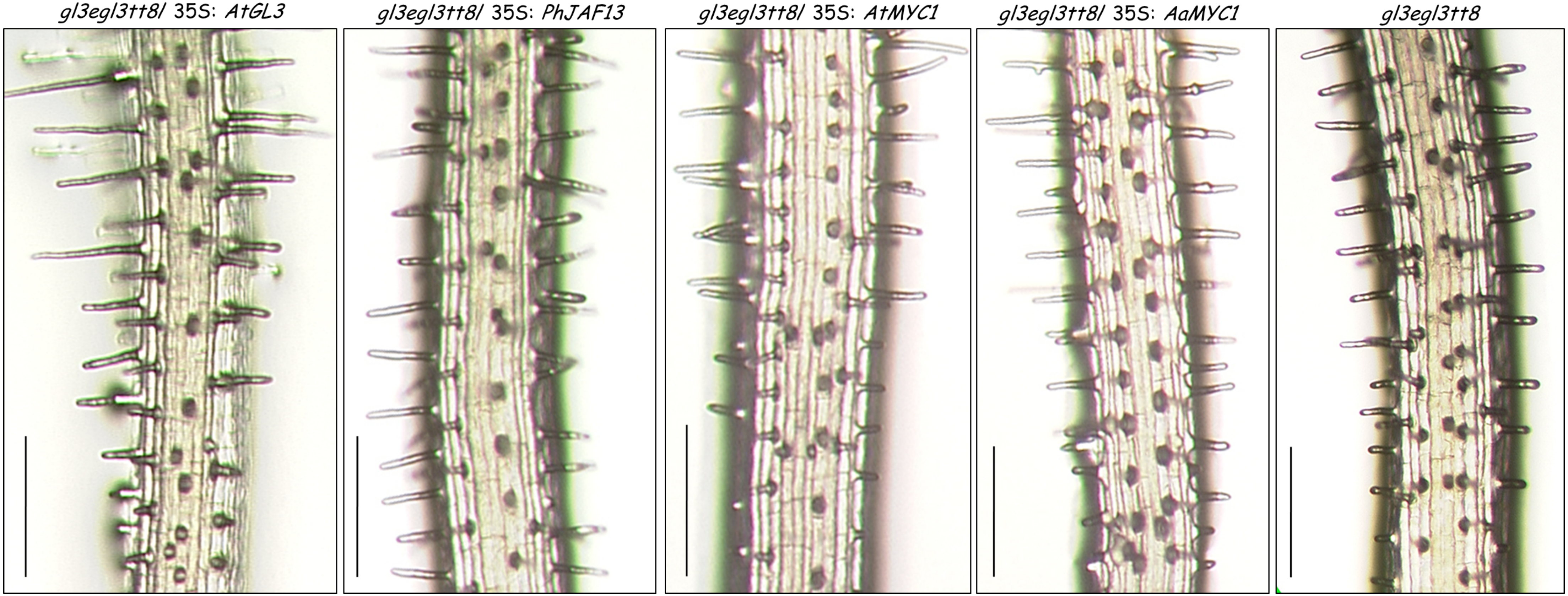

Figure S3. Root hair phenotype of *gl3egl3tt8* triple mutant rescued by 35S: *bHLHs* in T2 seedlings. Typically, the number of hairs in N-files are not completely suppressed, but clearly reduced in comparison to the triple mutant (Scale bar =200 **μ**m).

Figure S4

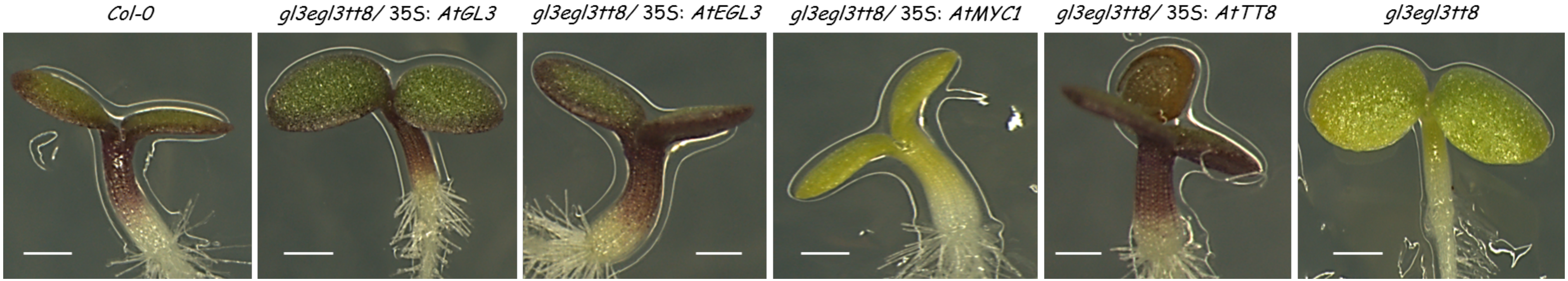

Figure S4. Anthocyanidin phenotype of *gl3egl3tt8* triple mutant rescued by 35S: *bHLHs* in T2 progeny. Anthocyanidin production in the hypocotyl is induced by 1/2MS with 3% sugar in wild type and in most rescued plants but not in the *gl3 egl3 tt8* mutant (Scale bar =2 mm).

Figure S5

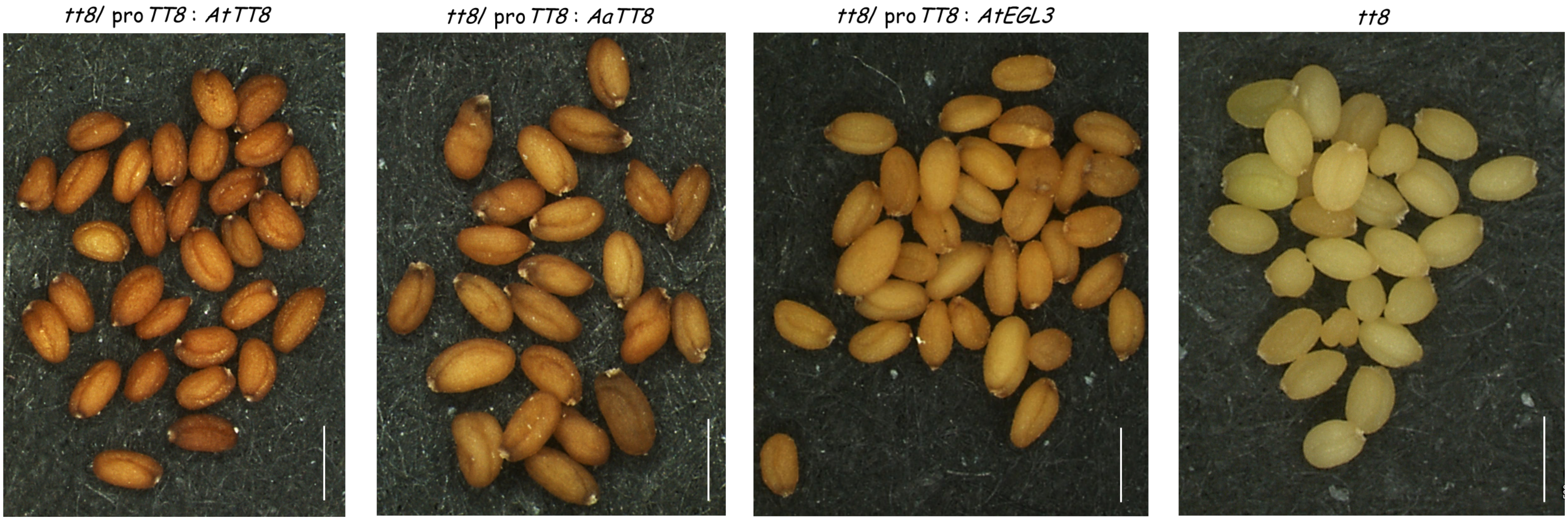

Figure S5. Seed coat color phenotype of *tt8* single mutant rescued by pro*TT8*: *bHLHs* in T2 progeny. Proanthocyanidin production can be seen by the brownish seed coat color that is absent in the *gl3 egl3 tt8* mutant. (Scale bar =100 **μ**m)

Figure S6

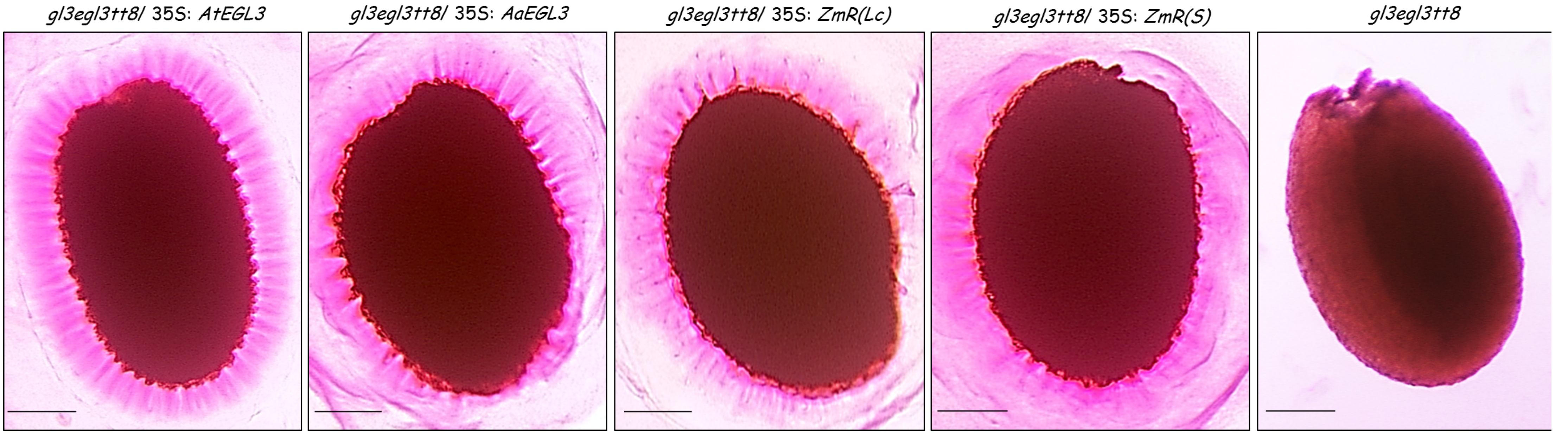

Figure S6. Seed coat mucilage phenotype of *gl3egl3tt8* triple mutant rescued by 35S: *bHLHs* in T2 progeny. Ruthenium red stains both seed coat mucilage layers. The inner mucilage layer is stained dark red, the outer light violet. The *gl3 egl3 tt8* mutant produces no mucilage (Scale bar =100 **μ**m).

Figure S7

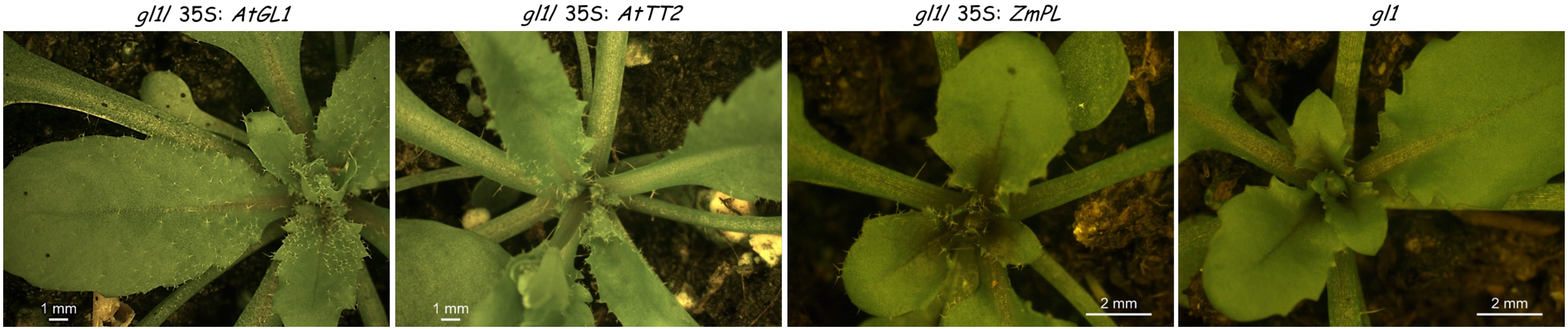

Figure S7. Trichome phenotype of *gl1* single mutant rescued by 35S:*R2R3MYBs* in 3-week-old T1 seedlings. Trichomes were observed on the basal part of true leaves of 35S:*AtGL1*. In *gl1* plants rescued with AtTT2 and ZmPL trichomes were found at the leaf margins.

Figure S8

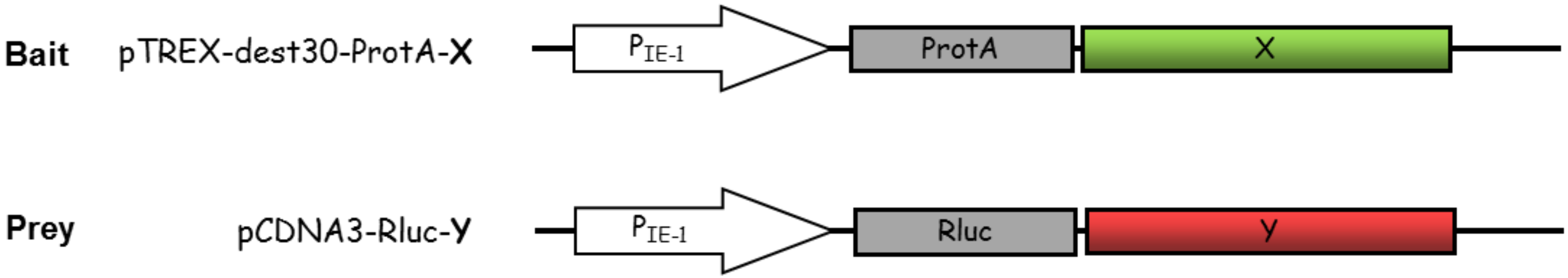

Figure S8. Schematic presentation of Human Promoter cytomegalovirus immediate early 1 (IE-1) driven constructs used in pairwise LUMIER assays.
Staphylococcus aureus protein A (ProtA) or with the *Renilla reniformis* luciferase (Rluc) are fused to N termini of bait proteins and prey proteins, respectively.
